# Label-free single-cell phenotyping to determine tumor cell heterogeneity in pancreatic cancer in real time

**DOI:** 10.1172/jci.insight.169105

**Published:** 2025-05-27

**Authors:** Katja Wittenzellner, Manuel Lengl, Stefan Röhrl, Carlo Maurer, Christian Klenk, Aristeidis Papargyriou, Laura Schmidleitner, Nicole Kabella, Akul Shastri, David E. Fresacher, Farid Harb, Nawal Hafez, Stefanie Bärthel, Daniele Lucarelli, Carmen Escorial-Iriarte, Felix Orben, Rupert Öllinger, Ellen Emken, Lisa Fricke, Joanna Madej, Patrick Wustrow, I. Ekin Demir, Helmut Friess, Tobias Lahmer, Roland M. Schmid, Roland Rad, Günter Schneider, Bernhard Kuster, Dieter Saur, Oliver Hayden, Klaus Diepold, Maximilian Reichert

**Affiliations:** 1 Translational Pancreatic Cancer Research Center and; 2 Technical University Munich (TUM) School of Medicine and Health, Department of Clinical Medicine – Clinical Department for Internal Medicine II, University Medical Center, TUM, Munich, Germany.; 3 Chair for Data Processing, TUM, Munich, Germany.; 4 Heinz Nixdorf Chair of Biomedical Electronics, TranslaTUM;; 5 Center for Protein Assemblies (CPA); and; 6 Center for Organoid Systems (COS), TUM, Garching, Germany.; 7 Bavarian Cancer Research Center (BZKF), Munich, Germany.; 8 Institute of Stem Cell Research, Helmholtz Center Munich, Neuherberg, Germany.; 9 Chair of Proteomics and Bioanalytics, TUM, Freising, Germany.; 10 Institute for Translational Cancer Research and Experimental Cancer Therapy, TranslaTUM, TUM, Munich, Germany.; 11 German Cancer Consortium (DKTK), partner site Munich, a partnership between German Cancer Research Center (DKFZ) and University Hospital Klinikum rechts der Isar, Munich, Germany.; 12 DKFZ and DKTK, Heidelberg, Germany.; 13 Department of Computational Health, Institute of Computational Biology, Helmholtz, Munich, Germany.; 14 Institute of Molecular Oncology and Functional Genomics, TranslaTUM, TUM School of Medicine, and; 15 Department of Surgery, University Medical Center, Klinikum rechts der Isar, TUM, Munich, Germany.; 16 Department of General, Visceral and Pediatric Surgery, University Medical Center Göttingen, Göttingen, Germany.

**Keywords:** Gastroenterology, Oncology, Cancer

## Abstract

Resistance to chemotherapy of pancreatic ductal adenocarcinoma (PDAC) is largely driven by intratumoral heterogeneity (ITH) due to tumor cell plasticity and clonal diversity. To develop alternative strategies to overcome this defined mechanism of resistance, tools to monitor and quantify ITH in a rapid and scalable fashion are needed urgently. Here, we employed label-free digital holographic microscopy (DHM) to characterize ITH in PDAC. We established a robust experimental and machine learning analysis pipeline to perform single-cell phenotyping based on DHM-derived phase images of PDAC cells in suspension. Importantly, we were able to detect dynamic changes in tumor cell differentiation and heterogeneity of distinct PDAC subtypes upon induction of epithelial-mesenchymal transition and under treatment-imposed pressure in murine and patient-derived model systems. This platform allowed us to assess phenotypic ITH in PDAC on a single-cell level in real time. Implementing this technology into the clinical workflow has the potential to fundamentally increase our understanding of tumor heterogeneity during evolution and treatment response.

## Abstract 2

Using label-free digital holographic microscopy, this study provides real-time single-cell insights into pancreatic cancer heterogeneity, aiding clinically relevant understanding of treatment-induced tumor cell reprogramming.

## Introduction

Pancreatic ductal adenocarcinoma (PDAC) belongs to the deadliest types of malignant tumors with an overall 5-year survival rate less than 11% despite a low incidence rate compared with other cancers (1). Polychemotherapies, such as FOLFIRINOX or gemcitabine/nab-paclitaxel (2, 3), as well as targeted therapies demonstrate limited efficacy in PDAC (4, 5). To improve patient outcomes, in the past decade, research has focused mainly on the characterization of PDAC on the molecular level. Extensive PDAC-subtyping efforts based on transcriptomic profiling revealed 2 major subtypes in human tumors, the classical and the quasi-mesenchymal/basal-like/squamous subtype, correlating with histopathological gradings and survival rates (6–8). Similarly, Mueller et al. characterized a large cohort of murine *Kras^G12D^*-mutated PDAC cells, which allowed a separation into the C2 and C1 cluster representing epithelial and mesenchymal phenotypes as well as biology of the disease (9). However, Topham et al. investigated the reliability of these subtyping methodologies and found that 1 out of 6 patients with PDAC cannot be assigned to either the classical or quasi-mesenchymal categories, indicating limitations of the approach for clinical practice (10). A possible explanation constitutes the pronounced intratumoral heterogeneity (ITH), which is not captured when using these common bulk subtyping approaches. ITH, however, is a key feature of PDAC and might be a leading cause of treatment failure and relapse after chemotherapy due to selective and plastic processes occurring. Single-cell technologies, such as single-cell RNA sequencing (scRNA-Seq), bypass this problem, as they allow researchers to analyze inter- and particularly intratumoral heterogeneity. Based on single-cell transcriptomic profiling, Peng et al. found various subpopulations in the malignant cell cluster of PDAC with differentially activated signaling pathways as well as transcription factors, resulting in a diverse proliferative and migratory behavior of the tumor (11). Additionally, scRNA-Seq of 15 PDAC patient specimens was performed, and classical and basal-like tumor cells were found to coexist within 13 out of 15 samples, suggesting an extensive degree of ITH in PDAC (12). In line with this study, Lin et al. detected epithelial and epithelial-mesenchymal transition (EMT^+^) tumor cells intratumorally in PDAC specimen and showed a strong correlation between EMT^+^ tumor cells and poor survival (13). Single-cell technologies are substantial contributions for understanding the tumor evolution and allow an in-depth molecular analysis of ITH. However, the considerable processing and analysis time as well as financial strains represent a relevant challenge implementing these procedures into clinical routine, not to mention the lack of a uniform and standardized analysis pipeline (10). Generating information about the tumor differentiation and detailed composition instantly has the potential in the future to make a deep impact upon clinical decision-making. Therefore, we established digital holographic microscopy (DHM) for high-content characterization of PDAC heterogeneity (static) and plasticity (dynamic). DHM is based on the interference of a reference and an object beam, resulting in recorded holograms, from which quantitative phase information is reassembled, providing abundant intracellular information (14). Coupling the DHM to a microfluidic system allows us to record and analyze individual tumor cells in suspension not in a stationary but in a high-throughput manner (15, 16). By processing the acquired images using our specifically developed analysis pipeline, we were able to cluster PDAC cells from distinct transcriptional subtypes according to their individual phenotypes and ITH. Importantly, we employed this technology to detect alterations in morphology by induction of EMT or in response to chemotherapy in a translational setting characterizing PDAC patient-derived organoids (PDOs). Overall, we present a technology that can be applied to virtually any kind of tissue sample or tumor model in single-cell resolution, harboring great potential to translate the rapidly evolving knowledge of ITH in PDAC into clinics.

## Results

### DHM-based phenotyping of distinct states of PDAC tumor cell differentiation.

**Figure 1:**
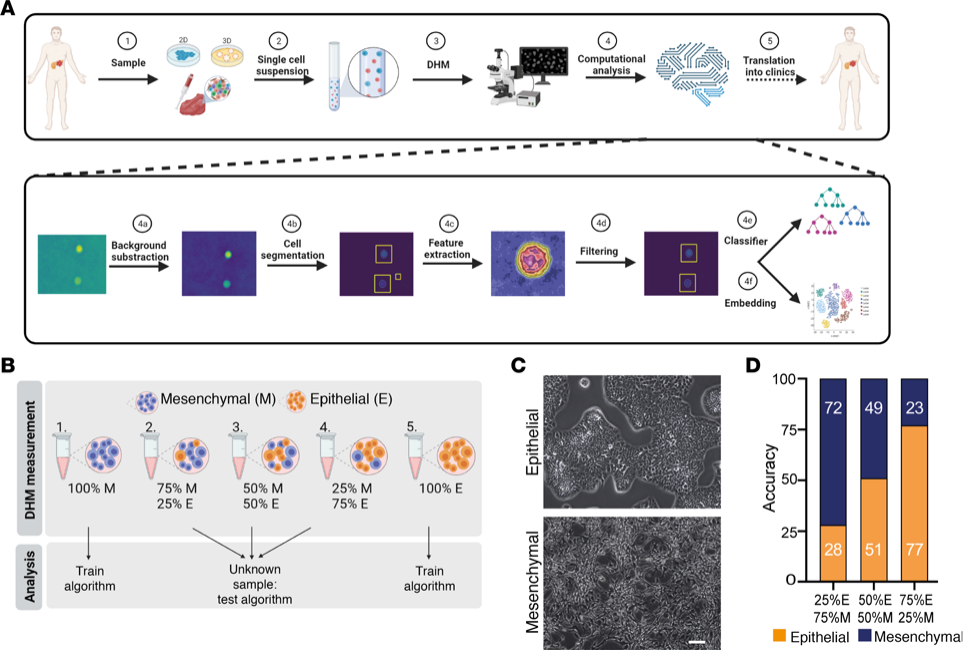
Establishing DHM-based single-cell phenotyping to detect tumor cell differentiation. (**A**) Schematic illustration of the established workflow and computational analysis pipeline. (**B**) Schematic illustration of the spike-in experiment setup. (**C**) Phase contrast images of epithelial (9591) and mesenchymal (16992) cells used in the spike-in experiment. Scale bar indicates 400 μm. (**D**) Accuracies obtained using random forest classification when trained with 100% epithelial and 100% mesenchymal PDAC cells.

The aim of this study was to establish DHM for a detailed phenotypic characterization of PDAC on a cellular level in a label-free, high-throughput fashion (Figure 1A). We used a customized setup, including a DHM that is coupled to a microfluidic system, allowing us to focus cells in suspension in flow for parallelized single-cell imaging. We dissociated PDAC cell cultures into a single-cell suspension and acquired single-cell–phase images in flow. The images were then subjected to a computational analysis pipeline. In the first step, we pre-processed the obtained phase images by subtracting the background to remove noise and artifacts. Then, using binary thresholding, we performed cell segmentation to find regions of interest. For feature extraction and filtering, we combined morphological features, partly derived from OpenCV (17) and partly in-house–created, with a lightweight residual neural network with 18 convolutional layers (ResNet18). In a classification step, we applied the commonly used classification tools random forest, support vector machine, K-nearest neighbors, and neural network in order to discriminate experimental samples and found the random forest classification to perform best throughout this study. Finally, we applied hierarchical clustering to demonstrate relationships between experimental samples and uniform manifold approximation and projection (UMAP) clustering to further visualize all single data points in a spectrum ranging from epithelial to mesenchymal.

To use DHM as a tool to investigate the phenotype of an unknown sample, it is key to identify different morphological subpopulations within one sample. Thus, we first performed a spike-in experiment, mixing a characterized epithelial (9591) as well as a mesenchymal cell line (16992) (9), both derived from a *Kras^G12D^*-driven PDAC mouse model, in defined ratios (Figure 1, B and C). The algorithm was trained using pure populations of both lines, and the mixed populations were tested afterward. The results of the DHM measurement substantially correlated with the calculated concentrations with a maximum deviation of 3% in the sample consisting of 25% epithelial and 75% mesenchymal cells (Figure 1D).

These data clearly demonstrate the robustness of this technique to identify subpopulations of varying morphologies within one sample. This in turn is an important factor, as ITH is highly abundant in PDAC, which can be determined on a single-cell level using our DHM approach. In addition, the approach is applicable to samples previously unseen by the algorithm.

### Single-cell phenotyping of PDAC tumor cells upon induction of EMT.

**Figure 2:**
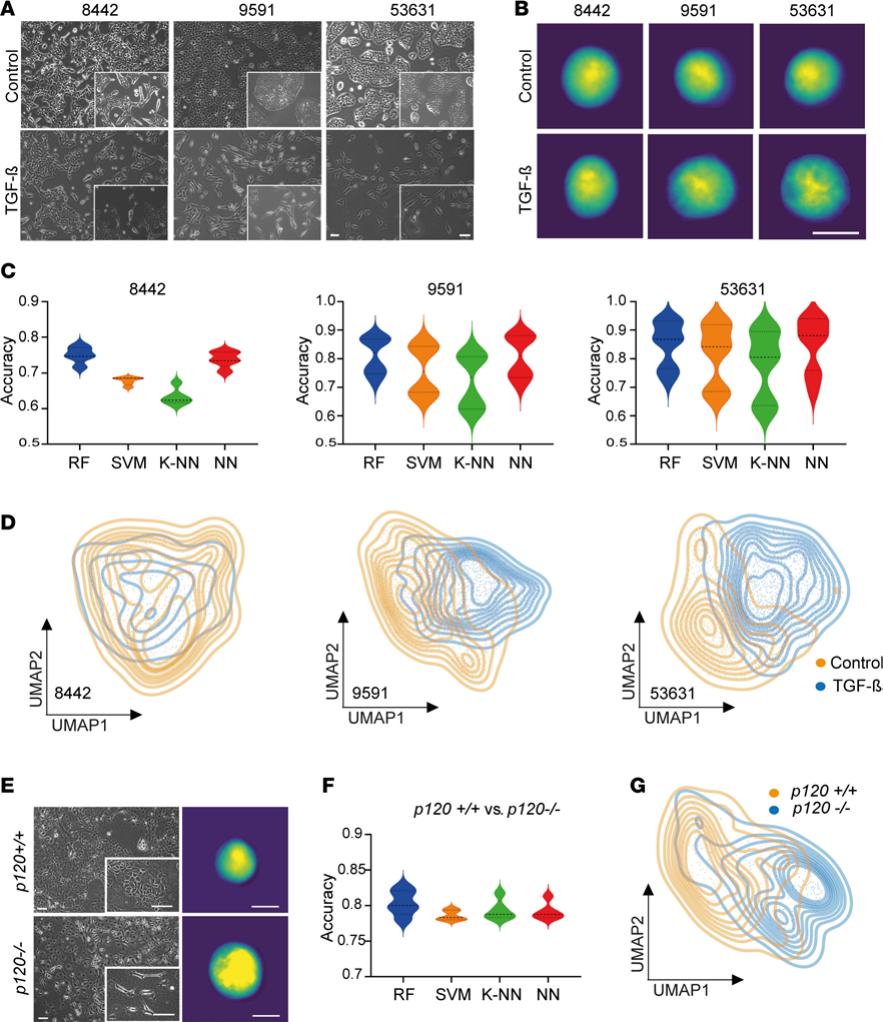
DHM-based identification of TGF-β– and genetically induced EMT. (**A**) Phase contrast images of control and TGF-β–treated epithelial PDAC cells. Scale bars represent 200 μm. (**B**) Representative DHM phase images in false colors of control and TGF-β–treated PDAC cells in suspension. Scale bar represents 10 μm. (**C**) Accuracy for separating control and TGF-β–treated PDAC cells individually for every cell line using different classification methods: random forest (RF), support vector machine (SVM), k-nearest neighbors (K-NN), and neural network (NN). Shown are the median and upper and lower quartiles. (**D**) Unsupervised clustering of control and TGF-β–treated PDAC cells based on DHM phase images and visualized using UMAP plots. (**E**) Representative phase contrast (left) and DHM phase images in false colors (right) of cells with *p120catenin* wild-type (*p120^+/+^*) or homozygous (*p120^–/–^*) deletion. Scale bars represent 200 μm (left) and 10 μm (right). (**F**) Accuracy for separating *p120^+/+^* and *p120^–/–^* cells using different classification methods as in **C**. Shown are the median and upper and lower quartiles. (**G**) Unsupervised clustering of *p120^+/+^* and *p120^–/–^* cells based on DHM phase images and visualized using UMAP plots.

As an experimental system to detect the whole spectrum of tumor cell dedifferentiation, we used TGF-β–induced EMT. Specifically, we exposed epithelial murine PDAC cells (8442, 9591, and 53631) to TGF-β for 14 days. As expected, when exposed to TGF-β, adherent epithelial tumor cells grown on culture dishes 2-dimensionally (in 2D) underwent EMT visible in classical phase contrast microscopy (Figure 2A) by losing cell-to-cell contacts and acquiring a spindle-shape morphology to a varying degree (Supplemental Figure 1A; supplemental material available online with this article; https://doi.org/10.1172/jci.insight.169105DS1). In addition, we assessed EMT markers of untreated as well as TGF-β–treated cells on mRNA as well as protein levels. In line with changes in morphology, a decrease in the epithelial marker E-cadherin and an increase in the mesenchymal markers N-cadherin and vimentin were observed (Supplemental Figure 1, A and B, and Supporting Data Values sheet 1). Notably, compared with PDAC cell lines 9591 and 53631, PDAC cell line 8442 did not respond to TGF-β treatment markedly, neither morphologically as depicted in the phase contrast images nor on mRNA and protein levels of indicated EMT markers (Supplemental Figure 1, A and B).

We next applied DHM to analyze single-cell EMT phenotypes (Figure 2B). As indicated in Figure 2C, we clearly detected differences in the respective DHM phase images. For cell lines 9591 and 53631, the algorithm was able to distinguish untreated controls and TGF-β–treated cells with an accuracy of 81% and 85%, respectively (Figure 2C and Supporting Data Values sheet 2). In contrast, line 8442, which showed only subtle changes upon TGF-β exposure, showed an accuracy of 75% on a single-cell phenotypic level (Figure 2C and Supporting Data Values sheet 2). Using UMAP clustering, a clear right shift (E to M) was identified in 9591 and 53631, whereas 8442 showed a minor transition with a massive overlap of control and TGF-β–treated cellular phenotypes (Figure 2D). This, in turn, recapitulated our observations of line 8442 in the validation experiments, assessing EMT by immunofluorescence, and was taken as an internal quality control for the algorithm we used in this study. To further test the transferability of our system, we trained and tested the algorithm across different cell lines. We limited the training of the algorithm to images of the cell line 53631 and evaluated its performance in discriminating treated and untreated cells of the unknown cell lines 8442 and 9591 (Supplemental Figure 1C). Using UMAP clustering, we obtained highly similar results as when trained on the identical cell line, showing the applicability of this technology for unknown samples. To validate the observed broad-range accuracy in DHM analyses (Figure 2C) and the shift in UMAP clustering (Figure 2D) as indicators of intra–cell line heterogeneity and EMT program induction, we conducted fluorescence-activated cell sorting (FACS) analysis to quantify the distribution of EMT markers N-cadherin (M), vimentin (M), E-cadherin (E), and EpCAM (E) in both control and TGF-β–treated conditions across 3 PDAC cell lines: 8442, 9591, and 53631 (Supplemental Figure 2). EMT induction was verified after 14 days of TGF-β exposure through changes in cell morphology observed in bright-field microscopy (Supplemental Figure 2A) and global marker protein expression (Supplemental Figure 2, B–D). Notably, UMAP visualizations of the FACS data revealed that subsets of tumor cells within each sample altered their marker expression in response to EMT induction, while a substantial subpopulation retained the marker profile indicative of their pretreatment state (Supplemental Figure 2D). This demonstrates inherent intra–cell line heterogeneity and the broad spectrum of tumor cell differentiation along the EMT axis within each PDAC line, supporting our DHM results.

In a complementary approach to study EMT, we used genetically engineered murine *Kras^G12D^*-mutant cells with either a wild-type *p120-catenin* (*p120ctn*) or homozygous deletion of *p120ctn*. Biallelic depletion of *p120ctn* leads to endocytosis and degradation of E-cadherin and thereby induces the loss of epithelial identity accompanied by acquired morphological features related to EMT (18). Consequently, genetically modulating epithelial plasticity via *p120ctn* was used as an alternative model system to train DHM for detecting different stages of EMT and plasticity important for PDAC characterization. The differences in phenotype upon *p120ctn* ablation were clearly visible in phase contrast microscopy (Figure 2E) as well as immunofluorescence (IF) staining of cells in an adherent condition, with the *p120ctn^–/–^* cells showing spindle-like structures (Supplemental Figure 1D). Thus, indirectly removing E-cadherin from the adherens junctions without an increase of the mesenchymal marker vimentin was enough to remodel cellular morphology (Supplemental Figure 1D). These morphologically different lines were subjected to DHM analysis as well (Figure 2E). Random forest classification allowed a separation of 80% between wild-type and knockout cells with a clear right shift of the cells harboring a *p120ctn* deletion shown in UMAP analysis (Figure 2, F and G, and Supporting Data Values sheet 3). Thus, solely by removing *p120ctn* without the increase of mesenchymal markers, a morphological shift could be detected in the majority of cells using DHM-based phenotyping.

Using DHM, we were able to detect TGF-β– and genetically induced EMT in murine PDAC cells in suspension. Importantly, our results indicate the ability of DHM to quantify the EMT spectrum of PDAC cells on a single-cell level.

### Detection and quantification of inter- and intratumor cell heterogeneity in murine and human models of PDAC subtypes.

**Figure 3:**
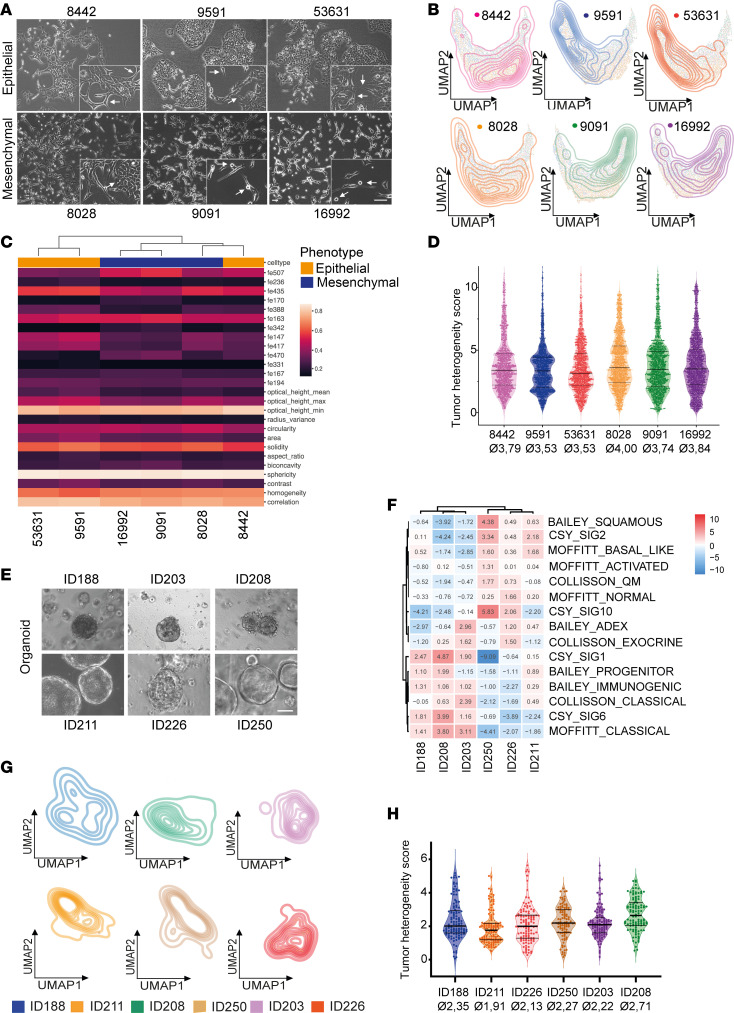
Single-cell phenotyping identifies heterogeneity in murine and human PDAC models. (**A**) Phase contrast images of epithelial and mesenchymal PDAC cells. Arrows indicate subpopulations of the opposing phenotype. Scale bars represent 200 μm. (**B**) Unsupervised clustering of individual cell lines based on DHM phase images visualized using UMAP plots. (**C**) Hierarchical clustering of DHM phase images derived from epithelial and mesenchymal PDAC cells based on the most different ResNet18 and morphological features. (**D**) Evaluation of intra–cell line heterogeneity using single-cell distance to cluster centroid. Kruskal-Wallis test, *P* < 0.0001. Shown are the median and upper and lower quartiles. Ø, mean tumor heterogeneity score. (**E**) Phase contrast images of patient-derived PDAC organoids. Scale bar represents 200 μm. (**F**) Molecular subtype classifier gene sets applied to transcriptomic profiles of PDOs. (**G**) Unsupervised clustering of individual PDAC organoid lines based on DHM phase images and visualized using UMAP plots. (**H**) Evaluation of intra–organoid line heterogeneity using single-cell distance to cluster centroid. Kruskal-Wallis test, *P* < 0.0001. Shown are the median and upper and lower quartiles.

After assessing single-cell phenotypes upon EMT induction, we focused on distinct transcriptional PDAC subtypes. To this end, we used molecularly characterized murine PDAC cells recently grouped into the C2b and C1 cluster representing epithelial and mesenchymal cell morphology, respectively (9). Hierarchical clustering based on their bulk transcriptomes (2,000 most differentially expressed genes) clearly separated them according to their defined transcriptomic cluster (Supplemental Figure 3A). Strikingly, we found a rather hybrid-like phenotype in 8442 with a large number of cells showing mesenchymal features and 8028 exhibiting a considerable subpopulation of epithelial cells in an adherent cell culture condition (Figure 3A). The other cell lines showed a subpopulation of the opposite phenotype as well but not to the same extent as 8442 and 8028. This, in turn, emphasizes the presence of tumoral heterogeneity detected by DHM, even though these lines clustered as clearly epithelial and mesenchymal based on bulk RNA sequencing, suggesting that bulk sequencing is not sufficiently detecting heterogeneity. To quantify tumor heterogeneity, we implemented a DHM-derived tumor heterogeneity score by measuring the single-cell distance to cluster centroid in UMAPs derived from each sample.

As expected, measuring these lines with DHM and analyzing it using morphological combined with ResNet18-based features did not predict a clear separation of phenotypes (Supplemental Figure 3, B and C). Random forest classification of epithelial (C2b cluster) versus mesenchymal (C1 cluster) cells showed a differentiation accuracy of only 72%, with a great overlap in UMAP clustering (Supplemental Figure 3, C and D, and Supporting Data Values sheet 4). However, cell line–specific UMAP clustering revealed a continuum of cellular phenotypes within each cluster as well as cell lines demonstrating high inter- and intratumoral heterogeneity (Figure 3B). While the vast majority of 9591 and 53631 clustered on the epithelial side of the spectrum (left), 8442 covered the whole spectrum, with the majority of cells being located in the middle, indicating a hybrid phenotype. Similar to this, 8028 showed a strong overlap with the epithelial phenotype, while 9091 and 16992 clustered mainly on the mesenchymal side of the spectrum (Figure 3B). Additionally, hierarchical clustering based on DHM showed a closer relationship between 8442 and 8028 than each cell line had to the members of their transcriptomic subtype, further strengthening the hybrid-like EMT state of both lines (Figure 3C). When assessing the tumor heterogeneity score of these lines, we indeed found 8442 and 8028 exhibited the highest heterogeneity score compared with the other members of their respective transcriptomic clusters (Figure 3D, Supplemental Figure 3E, and Supporting Data Values sheet 5). These data strongly indicate that even though cells are grouped into a defined tumor subtype based on their bulk transcriptomic profile, they can be composed of a highly heterogeneous cell population, which can be analyzed in detail using only single-cell technologies, such as DHM.

In parallel, we performed the same approach using human established and well-characterized PDAC cell lines. When cultured in 2D, PatuS and HPAC exhibited an epithelial morphology with colony formation and cell-cell contact, while PSN-1, DanG, and PatuT showed a spindle-like single-cell growth pattern (Supplemental Figure 4A). When applying the well-known PDAC subtyping methodologies to these cell lines, we did not obtain definite subtypes for all cell lines (Supplemental Figure 4B). While PatuS as well as PatuT and DanG were clustered into the classical and the quasi-mesenchymal PDAC subtype, respectively, HPAC and PSN-1 failed to be assigned to either subtype, suggesting either a hybrid-like phenotype or a failure of subtyping approaches as Topham et al. demonstrated (10). However, when we measured these cells with DHM, we obtained a clear separation of the cells with epithelial and quasi-mesenchymal morphology (Supplemental Figure 4C). Hierarchical clustering based on DHM phase images clearly separated HPAC and PatuS from PatuT, PSN-1, and DanG, similar to the morphology observed in adherent cell culture (Supplemental Figure 4D). Random forest classification and UMAP clustering further verified this grouping, as the epithelial and quasi-mesenchymal cells could be separated with an accuracy of almost 90% (Supplemental Figure 4, E and F, and Supporting Data Values sheet 6). Interestingly, levels of intra–cell line heterogeneity differed independently of cellular morphology, with PatuS and PatuT showing the lowest and PSN-1 showing the highest tumor heterogeneity score (Supplemental Figure 4, G–I, and Supporting Data Values sheet 7).

In the past decade, 3D model systems, such as PDO cultures, have been extensively studied, since they more closely recapitulate human physiology and better depict heterogeneity compared with 2D cell cultures (19). Indeed, Juiz et al. performed scRNA-Seq of 6 PDAC PDO lines and grouped their transcriptomic profiles into 4 clusters illustrating different levels of EMT. Interestingly, these clusters were found throughout the whole cell line panel. However, the cell count of each cluster was different between the PDO lines, representing a high degree of ITH in these organoids (20). Therefore, we used in the next step 6 different PDO lines in order to identify inter- and intratumoral heterogeneity using DHM in a clinically relevant setting. While ID188, ID203, ID208, and ID226 showed a lumen-filling organoid growth pattern, ID211 and ID250 grew with hollow lumen (Figure 3E). Transcriptional subtyping clustered them into 2 groups; however, a clear classical subtype was present only in ID211 and a quasi-mesenchymal subtype only in ID250. For the rest of the lines, no definite subtype could be assigned (Figure 3F). DHM-based phenotyping did not predict a clear separation of the PDO lines into classical and quasi-mesenchymal using hierarchical clustering (Supplemental Figure 5, A and B), but rather demonstrated high inter- and intratumoral heterogeneity in organoid line–specific UMAP analysis (Figure 3G). Indeed, when quantifying the intra–cell line heterogeneity, we found significantly different levels in the 6 lines. While ID211 and ID226 showed the lowest heterogeneity within their respective cluster, ID208 showed by far the highest tumoral heterogeneity (Figure 3H, Supplemental Figure 5C, and Supporting Data Values sheet 8).

Next, to expand our research beyond pure tumor cell populations, we conducted a series of experiments mixing PDAC tumor cells and cancer-associated fibroblasts (CAFs). Analyzing mixtures of murine epithelial or mesenchymal PDAC cells and murine CAFs, we observed a wide range of accuracy. Specifically, mesenchymal PDAC cells were almost indistinguishable from CAFs, a limitation also apparent in single-cell RNA data sets (Supplemental Figure 6A). However, in a patient-derived setting, analyzing PDOs with CAFs from the same patient, we achieved nearly 94% accuracy using random forest classification. This was accompanied by a substantial phenotypic shift in the UMAP analysis (Supplemental Figure 6, B–D, and Supporting Data Values sheet 9).

Our findings underscore the pronounced inter- and intratumor cell heterogeneity in both murine and human PDAC models, emphasizing the complexity of pancreatic cancer biology. Importantly, advanced imaging techniques, like DHM, reveal diverse cellular phenotypes within defined tumor subtypes and detect different cell lineages, particularly in patient-derived contexts.

### Monitoring dynamic changes in tumor heterogeneity upon chemotherapeutic treatment.

Previously, it has been shown that chemotherapeutic treatment such as FOLFIRINOX (FFX) or gemcitabine plus nab-paclitaxel can induce changes in PDAC subtype of primary tumors and metastasis by various processes, such as clonal selection or cellular plasticity (12, 21, 22). Here, we sought to track morphological changes upon FFX administration in distinct PDAC subtypes and PDOs using our DHM-based phenotyping approach.

**Figure 4:**
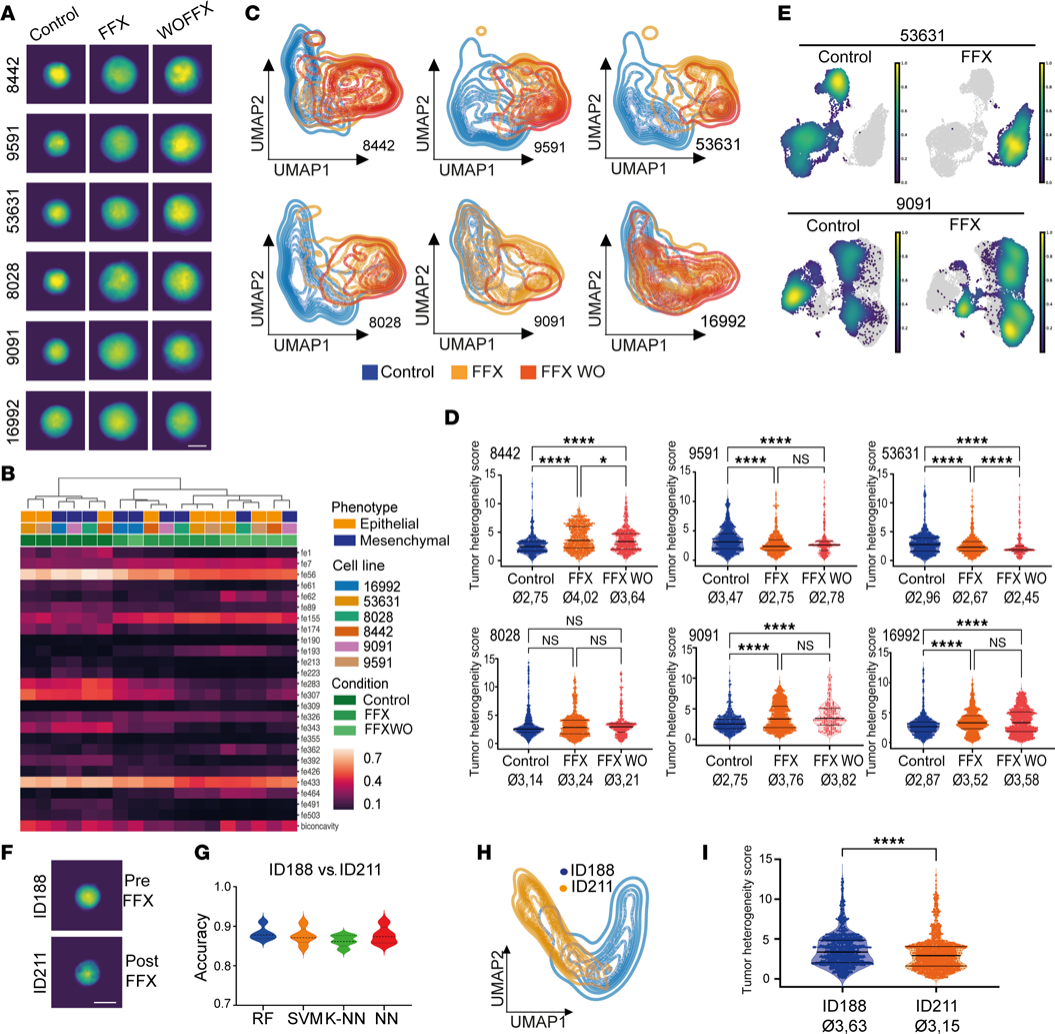
Monitoring single-cell phenotypes and heterogeneity in response to treatment. (**A**) Representative single-cell DHM phase images of untreated, FFX-treated, or FFX washout murine PDAC cells in suspension. Scale bar indicates 10 μm. (**B**) Hierarchical clustering of DHM phase images derived from murine PDAC cells in untreated, FFX-treated, or FFX washout condition based on the most different ResNet18 and morphological features. (**C**) Unsupervised clustering of different conditions in the individual cell lines based on DHM phase images and visualized using UMAP plots. (**D**) Evaluation of intra–cell line heterogeneity upon FFX treatment using single-cell distance to cluster centroid. Kruskal-Wallis test: *P < 0.05 and ****P < 0.0001. Shown are the median and upper and lower quartiles. (**E**) Kernel density of untreated and FFX-treated epithelial (53631) and mesenchymal (9091) cells analyzed using single-cell RNA-sequencing data. (**F**) Representative single-cell DHM phase images of pre– (ID188) and post– (ID211) FFX-treated PDOs in suspension. Scale bar indicates 10 μm. (**G**) Accuracy for separating organoids before and after FOLFIRINOX treatment using different classification methods: RF, SVM, K-NN, and NN. Shown are the median and upper and lower quartiles. (**H**) Unsupervised clustering of ID188 and ID211 organoids based on DHM phase images and visualized using UMAP plots. (**I**) Evaluation of intra–organoid line heterogeneity of ID188 and ID211 using single-cell distance to cluster centroid. Mann-Whitney test, ****P < 0.0001. Shown are the median and upper and lower quartiles.

First, we treated murine epithelial and mesenchymal PDAC cells for 72 hours with their respective IC_50_ of FFX, obtained by drug-response curves (Supplemental Figure 7A), and surviving cells were recovered for an additional 72 hours in a washout phase to differentiate temporary and prolonged effects. Transcriptomics, proteomics, as well as DHM-based phenotyping were performed in order to obtain detailed insight into FFX-induced molecular and phenotypic adaptations (Supplemental Figure 7B). Based on phase contrast images, epithelial cells responded phenotypically more strongly compared with mesenchymal cells, as they lost their cell-cell contact and acquired an EMT-like morphology (Supplemental Figure 7C). In contrast, mesenchymal cells retained their phenotypic characteristics (Supplemental Figure 7C). Hierarchical clustering based on DHM phase images revealed acquired similarities as well as differences between the cell lines upon FFX induction (Figure 4, A and B). We have already shown a close relationship between 8442 and 8028 with a rather hybrid-like phenotype in the control setting (Figure 3C); however, upon FFX treatment, both cell lines phenotypically drifted apart and acquired further phenotypic characteristics of the opposing transcriptomic subtype. While 8442 was tightly related to the mesenchymal line 9091 upon FFX treatment, 8028 behaved morphologically similarly to the epithelial lines 9591 and 53631, again validating them as outliers of their transcriptomic cluster (Figure 4B). Additionally, DHM enabled a differentiation between untreated and FFX-treated cells with an accuracy of roughly 87% when compared in a cell line– and phenotype-independent fashion using random forest classification (Supplemental Figure 7D and Supporting Data Values sheet 10). When they were visualized using UMAP clustering, we observed a clear right shift in the FFX and the FFX washout cells, indicating a certain degree of EMT occurring upon FFX treatment (Supplemental Figure 7E). Next, we performed a cell line–specific UMAP clustering to illustrate the single-cell behavior and EMT status of individual lines upon FFX administration (Figure 4C). While we detected a clear right shift toward a more mesenchymal phenotype for the lines 8442, 9591, 53631, and 8028 in the treated and washout condition, half of the population of 9091 and 16992 remained rather unaffected by FFX treatment. To validate these DHM results, we compared the single-sample EMT (ssEMT) score of the HALLMARK gene set retrieved from transcriptomic and proteomic profiling between the control, FFX, and FFX washout conditions. Individual cell lines exhibited different levels of EMT upon FFX treatment (Supplemental Figure 7F and Supporting Data Values sheet 11). On the mRNA level, 53631 showed the highest change in the EMT score of the epithelial lines, which was detected in the cell line–specific UMAP clustering, as well. Interestingly, the mesenchymal line 16992 showed a significant increase in the EMT score upon FFX as well, which, however, cannot be fully confirmed on a single-cell level, as half of the population remained rather unaffected. On a protein level, the effects of FFX treatment on the EMT score were augmented in all lines. Nevertheless, 53631 and 16992 were the lines with the most significant change in the ssEMT score upon FFX in their respective transcriptomic cluster. Interestingly, when analyzing the ITH score by calculating the single-cell distance to the cluster centroid, we found a subtype-dependent change in ITH upon FFX administration (Figure 4D and Supporting Data Values sheet 12). While the ITH significantly decreased in the epithelial lines 9591 and 53631 in the FFX and the washout cells, it significantly increased in the mesenchymal lines 9091 and 16992 upon chemotherapy. Again, the line 8442 behaved similarly to the mesenchymal lines, showing an increase in ITH upon FFX compared with its untreated controls, again proving its outlier character.

To validate the phenotypic changes induced by FFX exposure, we conducted scRNA-Seq on a representative epithelial (53631) and mesenchymal (9091) PDAC cell line under both control and FFX treatment conditions (Figure 4E). Consistent with the DHM results, UMAP plots displayed pronounced reorganization of cell clusters when treated with respective FFX-IC_50_ concentrations. Specifically, epithelial PDAC cells showed the emergence of a new and consolidated cluster, characterized by upregulated expression of genes such as mouse double minute 2; prostaglandin-endoperoxide synthase 2, also known as COX-2; and cyclin G1, which are involved in cell cycle regulation, apoptosis, and inflammation (Figure 4E and Supplemental Figure 8A). In contrast, and in alignment with DHM-based UMAP clustering, FFX treatment in mesenchymal PDAC cells induced a subtle shift in the relative proportions of cells within preexisting clusters. This was accompanied by increased expression of genes like tolloid-like 1 and cadherin 9, associated with extracellular matrix remodeling and cell-cell adhesion (Figure 4E and Supplemental Figure 8B).

To apply this technology in a clinically relevant setting, we compared the PDO lines ID188 and ID211 in more detail as they were derived from the same patient with PDAC before and after neoadjuvant FFX therapy. While ID188 was established from a treatment-naive fine-needle biopsy (FNB), ID211 was isolated from a surgical resection after 4 cycles of FFX. Recently, we have performed an in-depth characterization of this matched pair of PDOs on a molecular and functional level (22). Specifically, both lines were classified as classical PDAC subtype according to PurIST subtyping; however, we identified a certain degree of redifferentiation in ID211 upon FFX treatment, which was accompanied by a downregulation of pathways that are associated with the basal-like subtype such as KRAS and TGF-β signaling, cell cycle, hypoxia, as well as inflammation (22). When subjecting these lines to a 1-to-1 DHM comparison, we were able to phenotypically separate the 2 lines with an accuracy of 90% using random forest classification, though they were derived from the same patient (Figure 4, F and G, and Supporting Data Values sheet 13). Additionally, we could observe a left shift toward a more epithelial phenotype in the ID211 after FFX administration compared with ID188, verifying the previously shown process of redifferentiation (Figure 4H) (22). Importantly, we were able to identify a chemotherapy-induced reduction of ITH in ID211 compared with ID188 (Figure 4I and Supporting Data Values sheet 14), similar to what we observed in the murine epithelial PDAC cells upon FFX administration. In summary, by DHM-based phenotyping we are able to capture dynamic changes in differentiation occurring under chemotherapy on a single-cell level. Importantly, the sample acquisition and analysis pipeline are able to provide detailed information on the cellular heterogeneity of tumor samples, such as PDAC cell lines and PDOs and suspension of multiple cell lineages (PDOs and CAFs), in real time.

## Discussion

Identifying transcriptional subtypes of tumors and their EMT differentiation status was a main focus of PDAC research in the past decade, as these features correlate with treatment response and overall survival (6–8, 12, 23, 24). Multisampling of individual tumors as well as scRNA-Seq revealed a coexistence of distinct PDAC subtypes within the same tumor and a high degree of ITH (11–13). Importantly, ITH — genetic or epigenetic — is considered a major driver of therapy resistance in human cancer (25–27), and the pronounced ITH found in PDAC might be a leading cause for the lowest survival rate of PDAC compared with other cancers.

At the same time, single-cell profiling technologies, such as scRNA-Seq, remain challenging to be implemented in a scalable and clinically meaningful setting (28, 29).

Therefore, we established DHM as a tool for label-free phenotyping of PDAC cells, including detection of EMT on a single-cell level and quantification of inter- and intratumoral heterogeneity in real time using machine learning algorithms. Capturing the single-cell phenotype, which is the sum of different cellular processes occurring, for instance, on the genomic, transcriptomic, proteomic, and posttranslational levels, allows us to perform an unbiased and standardized phenotyping. Also, by introducing the ITH score, we provide a quantitative biomarker to study ITH in immediate and dynamic conditions in PDAC.

As a first step, we tested our system by genetically and pharmacologically inducing EMT in PDAC cells to correctly identify distinct stages of cellular differentiation. Using DHM allowed us to further phenotype numerous characterized epithelial and mesenchymal PDAC cells of human and murine origin regarding their EMT status on a single-cell level, which revealed a high degree of ITH. Applying this technique to primary patient-derived models such as PDOs enabled to characterize intratumoral heterogeneity in a clinically relevant model system. Importantly, the presence of heterogeneity within different PDO lines was recently demonstrated using single-cell transcriptomics (20, 30). In line with our results, both of these studies demonstrate that PDOs assigned to the classical subtype, indeed, harbor cell clusters of the basal-like phenotype (20, 30). Importantly, basal-like differentiation is associated with increased resistance toward chemotherapy (24, 30). Therefore, real-time single-cell phenotyping harbors great potential for monitoring response to treatment in a clinical setting, especially in highly plastic and heterogeneous tumors, such as PDAC.

In this study, we focused on EMT plasticity and quantification of cellular heterogeneity within the tumor cell compartment of PDAC. This technology offers a wide range of future applications in PDAC research and clinical care and can be expanded to additional cellular compartments within the tumor microenvironment. For example, CAFs display remarkable plasticity, which substantially affects PDAC biology (31). Similarly, distinct subregions within the TME harbor distinct tumor-promoting and chemoprotective functions and are correlated with patient outcome (32). Therefore, gaining immediate insight into the cellular composition of tumors by single-cell phenotyping has the potential to change current diagnostics in PDAC and bridge the implementation gap of complementary single-cell technologies, such as scRNA-Seq.

In addition, we and others have previously shown that tumor cells in PDAC display remarkable plasticity under chemotherapeutic pressure (22, 33). Indeed, in murine model systems, we identified a phenotypic shift from baseline upon FFX treatment to a different extent depending on the cell line and its baseline morphology using DHM. These results could be confirmed on a global gene expression level via scRNA-Seq. Additionally, PDOs derived from the same patient with PDAC before and after FFX treatment display a phenotype switch, in this case mesenchymal-epithelial transition and reduced ITH. Importantly, the phenotype of posttreatment organoids was linked to a specific therapeutic vulnerability toward MEK inhibition (22).

In summary, capturing tumor cell differentiation and dynamic cell fate decision in a scalable fashion on a single-cell level offers the unique opportunity to learn how to perturb and therapeutically exploit these adaptive biological processes conferring resistance in PDAC.

## Methods

### Sex as a biological variable

Our study examined PDOs from both male and female patients with PDAC. The same applies to the murine PDAC cell lines, which were derived from male and female mice. However, the sex was not considered as a biological variable.

### Generation and culture of PDOs

PDOs were generated from EUS-guided fine needle aspiration/biopsy and surgical resection as described (34). Briefly, biopsies were minced and surgery specimens were incubated rotating for collagen digestion using a digestion buffer: DMEM-F12 (31330095, Thermo Fisher Scientific), 1× Primocin (ant-pm-2, InvivoGen), and 6 mg/mL collagenase II (17101015, Thermo Fisher Scientific). Tissue pellets were incubated for 3–10 minutes with RBC lysis buffer (A1049201, Thermo Fisher Scientific) and further digested using TrypLE (12604039, Thermo Fisher Scientific). Cell pellets were resuspended in 50 μL of Matrigel/well (354230, Corning Life Sciences), and PDO medium was added 10 minutes later after Matrigel was solidified: DMEM-F12; 5 mg/mL d-glucose (G8270, Sigma-Aldrich); 0.5% ITS Premix (354350, Thermo Fisher Scientific); 5 nM 3,3,5-triiodo-l-thyronine (T0821, Sigma-Aldrich); 1 μM dexamethasone (D175, Sigma-Aldrich); 100 ng/mL cholera toxin (C9903, Sigma-Aldrich); 1% penicillin/streptomycin (15140122, Thermo Fisher Scientific); 5% NU-Serum IV (355500, Thermo Fisher Scientific); 25 μg/mL bovine pituitary extract (P1167, Sigma-Aldrich); 10 mM nicotinamide (N3376, Sigma-Aldrich); 100 μg/mL Primocin (ant-pm05, InvivoGen); 0.5 μm A83-01 (2939, Tocris); 10% RSPO1-conditioned medium (R-spondin-1–overexpressing cell line HEK293T, provided by the Hubrecht Institute, Utrecht, the Netherlands); 100 ng/mL Recombinant Human Heregulin-1 (100-03, PeproTech); and 10 μM Rho Kinase Inhibitor (TB1254-GMP, Tocris). For passaging, media were aspirated, and 250 μL Cell Recovery Solution (11543560, Thermo Fisher Scientific) was added to wells for 5 minutes. Mixture was dissolved in 1 mL ice-cold PBS (14190144, Thermo Fisher Scientific) supplemented with 0.1% BSA (11930, Serva). After 30 minutes on ice, organoids were centrifuged at 1,000 rpm at 4°C for 5 minutes, washed, and centrifuged again. Cell pellets were resuspended in 50 μL Matrigel/well, and medium was added 10 minutes later.

### 2D cell culture

Murine PDAC cells with *Kras^G12D^* mutation sourced in-house were cultured as described (9). Human PDAC cells sourced in-house were cultured in DMEM (41966052, Gibco) or RPMI medium (11875085, Gibco) supplemented with 10% fetal calf serum (10270106, Gibco) and 1% penicillin/streptomycin (1741838, Gibco) depending on cell line: DMEM: PaTu8988T (RRID:CVCL_1847), PaTu8988S (RRID: CVCL_1846); RPMI: DanG (RRID: CVCL_0243), HPAC (RRID:CVCL_3517), PSN-1 (RRID:CVCL_1644). Cells were authenticated regularly (October 2019) by Multiplexion GmbH or Microsynth AG. Passaging was performed using 0.05% trypsin-EDTA (15400054, Gibco), and cells were maintained at 37°C and 5% CO_2_.

For the TGF-β experiment, cell lines 8442, 9591, and 53631 were treated with a final concentration of 5 ng/mL recombinant TGFb1 (PeproTech, 100-21) for 14 days. Medium was changed every 2–3 days, and cells were split when reaching a confluence of maximum 80%.

For spike-in experiments, epithelial and mesenchymal PDAC cells as well as fibroblasts were cultured separately until DHM analysis. For measurement, cells were split, then precisely counted, and a final number of 1 × 10^6^ cells was mixed in the indicated ratios. After cell mixing, samples were analyzed on a single-cell level using DHM.

### In vitro FFX treatment

The 3-(4,5-dimethylthiazol-2-yl)-2,5 diphenyl tetrazolium bromide (MTT; M5655, Sigma-Aldrich) assay was used to determine cell viability after drug administration by measuring metabolic activity of cells. Therefore, 1,000 cells/well were seeded in 96-well plates as triplicates. For the in vitro FFX treatment, a mixture of 5-fluorouracil (C_max_ = 50 μM), irinotecan (C_max_ = 22.5 μM), and oxaliplatin (C_max_ = 10.6 μM) was prepared and added in a 7-point drug dilution for 72 hours. For MTT measurement, 10 μL of MTT reagent was added per well and incubated for 4 hours at 37°C. Afterward, cell medium was removed, and 200 μL of an ethanol-DMSO mixture (1:1) was added. After 15 minutes of shaking at room temperature (RT), absorption was measured at a wavelength of 595 nm using the Multiscan FC spectrophotometer (Thermo Fisher Scientific).

### IF staining

For IF staining, cells were washed and fixed with 4% paraformaldehyde (PFA) for 10 minutes at RT and treated with 0.15% glycine for 5 minutes followed by 2 minutes of permeabilization using 0.2% Triton X-100 in PBS. After washing, cells were blocked for 1.5 hours in 10% donkey serum and 0.1% BSA diluted in PBS. Primary antibodies were incubated overnight at 4°C in 0.1% BSA diluted in PBS. After washing, secondary antibodies were incubated for 2.5 hours at RT in the dark. Thereafter, cells were treated for 2 minutes with DAPI (0.03 μL/mL, D9542, Sigma-Aldrich), washed, and mounted. Slides were kept at 4°C until further imaging using the Leica TCS SP8 Confocal Microscope. Antibodies used are in Table 1.

### Quantitative real-time PCR

Cellular RNA was harvested using RLT buffer supplemented with 2-mercaptoethanol, and RNA isolation was done using RNeasy Mini Kit according to manufacturer’s protocol (74106, QIAGEN). A total of 1 μg RNA was transcribed into cDNA using SensiFast cDNA Synthesis Kit (BIO-65053, Bioline), and quantitative real-time PCR was performed with the SensiFast SYBR Hi-Rox Kit (BIO-92005, Bioline) on a StepOnePlus System (Applied Biosystems) following manufacturer’s instructions. Analysis was performed using the 2-ΔΔCt method. Primers are listed: mE-cadherin FW: 5′-TCAAGCTCGCGGATAACCAGAACA-3′ and RV: 5′-ATTCCCGCCTTCATGCAGTTGTTG-3′, mN-cadherin FW: 5′-ATGGCCTTTCAAACACAGCCACAG-3′ and RV: 5′-ACAATGACGTCCACCCTGTTCTCA-3′, hE-cadherin FW: 5′-GCCTCCTGAAAAGAGAGTGGAAG-3′ and RV: 5′-TGGCAGTGTCTCTCCAAATCCG-3′, hvimentin FW: 5′-AGGCAAAGCAGGAGTCCACTGA-3′ and RV: 5′-ATCTGGCGTTCCAGGGACTCAT-3′, mβ-actin FW: 5′-GTCGAGTCGCGTCCACC-3′ and RV: 5′-GTCATCCATGGCGAACTGGT-3′, hβ-actin FW: 5′-CACCATTGGCAATGAGCGGTTC-3′ and RV: 5′-AGGTCTTTGCGGATGTCCACGT-3′.

### Flow cytometry

Cell lines 8442, 9591, and 53631 were treated with recombinant TGFb1 for 14 days as described above. On day 14, cells were stained and analyzed by flow cytometry. In detail, single-cell suspensions were prepared and stained with Zombie UV fixable live-dead dye (BioLegend, 423107, 1:1,000) for 20 minutes. After washing with FACS buffer (1% BSA in PBS, 2 mM EDTA), cells were stained with antibody solution for cell surface marker staining for 30 minutes. After washing with FACS buffer, cells were fixed and permeabilized with eBioscience Intracellular Fixation & Permeabilization Buffer Set (Thermo Fisher Scientific, 88-8824-00) according to manufacturer’s instructions. Next, cells were stained with antibody solution for intracellular staining for 30 minutes. After washing with FACS buffer, cells were resuspended in FACS buffer and analyzed on a BD FACSAria Fusion. Compensation controls were prepared using Invitrogen UltraComp eBeads Plus Compensation Beads (Thermo Fisher Scientific, 01-3333-42). Live/dead controls were prepared using ArC Amine Reactive Compensation Bead Kit (Thermo Fisher Scientific, A10346). For analysis, samples were acquired using indicated marker combinations on a custom FACSAris Fusion cell sorter. Further downstream analysis of the data was performed using FlowJo V10 and OMIQ. Antibodies used were CD324 (E-Cadherin, DECMA-1; 1:50; Thermo Fisher Scientific 53-3249-82), Alexa Fluor 488 CD326 (EpCAM, G8.8-PE; 1:200; Thermo Fisher Scientific 12-5791-82), N-cadherin (13A9, Alexa Fluor 750; 1:20; Novus Biologicals NBP1-48309AF750), and vimentin (W16220A Alexa Fluor 647; 1:100; BioLegend 699307).

### RNA sequencing

RNA of murine PDAC cells and patient-derived 3D organoids was harvested for RNA sequencing using the RNeasy Mini Kit (QIAGEN) and RNeasy Micro Kit (QIAGEN) according to the manufacturer’s protocol. Library preparation for bulk sequencing of poly(A)-RNA was done as described (35). Barcoded cDNA of each sample was generated with a Maxima RT polymerase (Thermo Fisher Scientific) using oligo-dT primer containing barcodes, unique molecular identifiers (UMIs), and an adaptor. Ends of cDNAs were extended by a template switch oligo (TSO), and full-length cDNA was amplified with primers binding to the TSO site and the adaptor. New England BioLabs Ultra II FS kit was used to fragment cDNA. After end repair and A-tailing a TruSeq adapter was ligated, and 3′-end fragments were finally amplified using primers with Illumina P5 and P7 overhangs. In comparison with Parekh et al. (35), the P5 and P7 sites were exchanged to allow sequencing of the cDNA in read1 and barcodes and UMIs in read2 to achieve a better cluster recognition. The library was sequenced on a NextSeq 500 (Illumina) with 67 cycles for the cDNA in read1 and 16 cycles for the barcodes and UMIs in read2. Data were processed using the published Drop-seq pipeline (v1.0) to generate sample- and gene-wise UMI tables (36). Reference genome (GRCh38) was used for alignment. Transcript and gene definitions were used according to GENCODE version M25.

### Proteomics

#### Sample preparation.

Triplicates of each line and condition (control, FFX treated, FFX washout) were lysed using 200 μL lysis buffer (2% SDS in 40 mM Tris-HCl, pH 7.6). To hydrolyze DNA and reduce viscosity, sample was boiled at 95°C for 10 minutes, and trifluoroacetic acid was added to a final concentration of 1%, incubated for 1–2 minutes at 95°C, and subsequently quenched with *N*-methylmorpholine (final concentration of 2%) to obtain a pH of 7.5–8. Protein concentration in cell lysate was determined using the Pierce BCA Protein Assay Kit according to manufacturer’s protocol. In order to remove detergent from samples, protein lysate was processed via protein aggregation capture sample workup as described with minor modifications (37). Therefore, a bead suspension was prepared by mixing magnetic SeraMag-A (GE45152105050250, GE Healthcare, now Cytiva; c = 50 mg/mL) and SeraMag-B (GE65152105050250, GE Healthcare, c = 50 mg/mL) beads in a ratio of 1:1 and immobilized on a magnet and supernatant was removed. Beads were washed twice with 1 mL double-distilled H_2_O and resuspended in double-distilled H_2_O in the original volume. A total of 150 μg protein lysate was mixed 1:10 (protein/beads weight) with the bead suspension. Acetonitrile (ACN) was added to a final concentration of 70% and incubated at RT (18 minutes, 800 rpm). After discarding the supernatant, beads were washed twice using 1 mL 80% ethanol and once more with 1 mL of 100% ACN. For reduction and alkylation, beads were resuspended in 100 μL digestion buffer without trypsin [100 mM HEPES, 2 mM CaCl_2_, 55 mM 2-chloracetamid, 10 mM Tris-(2-carboxyethyl)-phosphin] and incubated for 1 hour at 37°C and 800 rpm. Proteins were digested overnight at 37°C and 1,000 rpm by adding trypsin 1:50 (trypsin/substrate weight) resuspended in 100 mM HEPES in a volume of 10 μL per sample. Digestion was quenched by adding formic acid (FA) to a final concentration of ~1%. Samples were sonicated 3 times for 30 seconds, centrifuged (5 minutes, 13,000*g*), and bead-immobilized on a magnet to collect the supernatant. Beads were washed with 50 μL double-distilled H_2_O, sonicated 3 times for 30 seconds, and centrifuged (5 minutes, 13,000*g*), and supernatants were combined with previous supernatants. Samples were frozen at –80°C and dried in a SpeedVac. Peptides were reconstituted in 500 μL 0.1% FA, desalted using tC18 RP solid-phase extraction cartridges (Waters Corp.; wash solvent: 0.1% FA; elution solvent: 0.1% FA in 50% ACN), frozen at –80°C, and dried in a SpeedVac. After desalting, samples were ready for measurement and stored at –20°C until liquid chromatography-tandem mass spectrometry (LC-MS/MS).

#### LC-MS/MS.

For microflow LC-MS/MS analysis, samples were analyzed on a microflow LC-MS/MS system as previously described using a Dionex UltiMate 3000 RSLCnano System equipped with a modified Vanquish pump (Thermo Fisher Scientific) coupled online to a Q Exactive Orbitrap HF-X mass spectrometer (Thermo Fisher Scientific) (38). Chromatographic separation was performed via direct sample injection onto a 15 cm Acclaim PepMap 100 C18 column (2 μm particle size, 11 mm inner diameter × 150 mm, Thermo Fisher Scientific) at a flow rate of 50 μL/min. Solvent A was 0.1% FA, 3% DMSO in double-distilled H_2_O, and solvent B was 0.1% FA, 3% DMSO in ACN. Samples were dissolved in 0.1% FA, and the equivalent of 50 μg of the protein digest was injected into the system.

Samples were separated with a gradient of 1% to 24% B in 105 minutes followed by an increase of B to 35% in 15 minutes. The HF-X was operated in data-dependent acquisition and positive-ionization mode using an optimized 28 Hz method (38). Full MS resolution was set to 120,000, and full MS automatic gain control (AGC) target was 3 × 10^6^ with a maximum injection time of 100 ms. Mass range was set to 360–1,300. MS2 spectra were recorded at 15,000 resolution. AGC target value for fragment spectra was set to 1 × 10^5^ with a maximum injection time of 22 ms. The dynamic exclusion duration was set to 40 seconds. The TopN algorithm value (39) was set to 50. For MS2 spectra, the minimum AGC target was kept at 2 × 10^3^. The isolation width was set to 1.3 *m/z*, and the first mass was fixed at 100 *m/z*. The normalized collision energy was set to 28%. MS1 and MS2 spectra were acquired in profile and centroid mode, respectively (38).

#### Data analysis.

Protein/peptide identification and quantification were performed using MaxQuant (v1.6.2.10) (40) by searching MS2 data against all protein sequences (canonical and isoforms) as annotated in the SwissProt reference database (mouse proteins only, 25,333 entries, downloaded December 17, 2020) using the embedded search engine Andromeda (41). All treatments and replicates were searched together. Carbamidomethylated cysteine was set as fixed modification. Oxidation of methionine and N-terminal protein acetylation were set as variable modification. Trypsin/P was specified as the proteolytic enzyme, and up to 2 missed cleavage sites were allowed. The FDR was set at 1% on peptide-spectrum match and protein levels. The match-between-runs feature and label-free quantification were enabled (42). All other settings were set to standard MaxQuant default settings.

### Bioinformatic analysis of transcriptomic and proteomic data

High-throughput mRNA gene expression data from indicated conditions were analyzed using R environment for statistical computing (v4.0.4). Proteomic data analysis was performed using the Perseus software (v1.6.14.0) and R (v 4.0.02) on identified and quantified protein groups using label-free quantification intensity values, which were filtered for contaminants and reverse hits. The data were normalized using median centering after log_2_ transformation.

For selected human PDO cultures and human 2D cell lines, respectively, molecular subtype classifier gene sets (6–8, 12) were scored per sample using analytic rank-based enrichment analysis (aREA) (43) after computing transcriptome-wide expression single-sample signatures first rank transforming and rescaling first each column (cell line sample) and then each row (gene) between 0 and 1. The resulting normalized enrichment score (NES) matrix with classifier sets in rows and individual cell lines in columns was illustrated using the pheatmap R package (44).

To determine single-sample NES regarding EMT, aREA was applied on normalized RNA-sequencing count and protein expression data, respectively, using HALLMARK_EPITHELIAL_MESENCHYMAL_TRANSITION gene set without rescaling genes before single-sample enrichment analysis. ssEMT scores were rescaled between 0 and 1 for better comparability between results from RNA-Seq and protein expression samples, respectively.

### scRNA-Seq

For scRNA-Seq, lines ID53631 and ID9091 either as controls or after 72 hours of FFX treatment were used (45). Cells were trypsinized and counted after performing a dead cell removal (dead cell removal kit, Miltenyi Biotec). We loaded 30,000 single cells on a 10x Genomics Chromium Next GEM Chip G to create gel beads-in-emulsion. For barcoding and library preparation the Chromium Single Cell 3′ v3.1 chemistry was used according to manufacturer’s instructions (10x Genomics). Quality/size analysis of cDNAs and libraries was performed on an Agilent Bioanalyzer 2100 using HS DNA Kit (Agilent).

After quality control, libraries were pooled equimolarly and sequenced following standard configuration of 28/10/10/90. Libraries were sequenced in a NovaSeq X + 10B-100 flow cell (Illumina) following standard protocol.

ScRNA-Seq alignment was performed using 10x Genomics Cell Ranger v7.1.0 with mm10 as reference genome. Starting from the Cell Ranger output, analysis was performed using the SCANPY toolkit (46). Data were quality controlled. Specifically mean absolute deviation (MAD) was calculated for both number of detected transcripts and detected genes according to recently published single-cell best practices (47). Cells with MAD value above 5 were discarded, together with cells having more than 25% of mitochondrial gene counts. Doublets were detected and discarded using scrublet (48).

After quality control, 48,884 cells remained, with average count per cell of 30,355 transcripts and 4,308 genes. Raw counts were normalized and log-transformed.

Keeping cell lines separate, dimensionality reduction was performed using principal component analysis, computing the neighborhood graph over the first 10 principal components accounting for most of the variability, and then UMAP was computed (49).

### DHM

#### Image acquisition.

To distinguish between mesenchymal and epithelial cells, an imaging technique providing sufficient contrast is required. Thus, DHM from Ovizio Imaging Systems was customized based on a Mach-Zehnder off-axis interferometer setup. The microscope is equipped with a Nikon CFI LWD objective with 40× magnification and a numerical aperture of NA = 0.55, an Oslon PowerStar SLED (Osram) with a wavelength of λ = 528 nm, and a PointGrey Grasshopper GS3U332S4 camera, which takes 105 frames per second with an exposure time of 5 μs. The light beam from the SLED transmits the sample located in the back focal plane of the microscope objective. Afterward the beam is split with a grating filter into diffraction parts and a nondiffraction part. The diffracted part is further shifted in *x* and *y* direction compared with the reference. By recombination of the shifted parts and the reference, they can be interfered, resulting in a hologram, which is recorded on the camera. Finally, phase and amplitude images can be extracted from the hologram using common off-axis interferometer reconstruction algorithms (50, 51). A more detailed description of the setup and the working principle has been described before (15, 52, 53).

#### Microfluidic chip.

To measure cells in high throughput, DHM was used as an imaging flow cytometer by combining an imaging setup with a microfluidic chip. The channel has a height of 50 μm, a width of 500 μm, and a total length of 50,000 μm fabricated of poly(methyl methacrylate) (Fraunhofer ICT-IMM). Two microfluidic focusing methods were combined for precise alignment of a submonolayer of cells, thus eliminating the need for further adjustment of the focus after an initial setup. Hydrodynamic focusing could be achieved by using 5 inlets, containing 1 sample flow, 2 side flows (y-sheaths), and a top and a bottom flow (z-sheaths). Here, the flow rates were adjusted so that the lateral streams were fixed at 0.5 μL/s and all other streams (sample, top and bottom) at 0.2 μL/s. This leads to a total flow rate of 1.6 μL/s, which was adjusted with a neMESYS Base 120 syringe pump system with 5 modules (cetoni GmbH). Each slot was equipped with a 2.5 mL gas tight syringe (VWR). Furthermore, 0.9% of the polymer polyvinylpyrrolidone (PVP, 360 kDa) diluted in PBS was used as media for each of the 5 inlets to achieve viscoelastic focusing of the sample. Overall, the maximum Reynolds number during the measurements is in the single-digit range (Re ≈ 6.5), which implies that all measurements were performed in the laminar flow regime at low shear rates. Due to the stability of the method, no realignment of the focus during measurement is needed.

#### Sample preparation.

2D cells and organoids were processed to a single-cell suspension, and cell pellets were thoroughly resuspended in 500–1,000 μL 0.9% PVP solution depending on pellet size. The solution containing single cells was repeatedly injected into the microfluidic chip in volumes of approximately 100 μL.

### Computational analysis

#### Pre-processing.

Capturing 105 hologram images per second, a single measurement contains roughly 10,000 shots. The commercial OsOne Software (Ovizio) reconstructs phase information from these holograms using Poisson integration (15, 53). The software provides 512 pixel (px) by 384 px phase images (typically within an interval [-2,6 rad]) containing multiple cells. These images were preprocessed to obtain usable single-cell patches. Background subtraction was performed to remove noise and artifacts of the microfluidic channel. The background was estimated by median of *n* = 50 images. Due to the fixed alignment of lens, camera, light, and microfluidic channel, background is not changing during a single capture and is assumed as static, allowing us to perform the computation only once based on the first images. Cell segmentation was carried out to find regions of interest by applying binary thresholding to phase images with a threshold value of 0.8. From resulting binary images contours for each cell were extracted using the OpenCV *findContours* implementation of the algorithm proposed by Suzuki et al. (54). Each contour, which covers more than 30 px, was stored with its corresponding 96 px by 96 px image patch around the center. For feature extraction and filtering, each digitized cell was morphologically analyzed (15, 16) by employing established functions from OpenCV completed with in-house–created methods (Table 2). A lightweight ResNet18 was used for feature extraction (55). The network was pretrained on an ImageNet dataset and thereby extracting the most prominent structures within images.

#### Classification.

For classification, we applied 4 commonly used methods: random forest classification, support vector machine, k-nearest neighbor, and neural network.

Random forest is an ensemble classification method based on a multitude of decision trees automatically constructed using different fractions of given dataset (56). Here, the average of all trained decision trees was used, and the algorithm was limited to 100 individual decision trees without setting a bound for maximum depth of a single tree.

Support vector machine is a binary linear classification technique, which divides data into 2 classes using the best hyperplane decision boundary (57, 58). This decision boundary is obtained by maximizing the margin between the class border and data samples using kernel-trick to enable nonlinear separation. Soft margin was used, allowing some data points to violate the margin condition, resulting in penalization. Here, best performances were achieved with the one-against-one training procedure using a penalty parameter C = 1 and radial basis function as a kernel.

A k-nearest neighbor classifier assumes that classes can be represented as distinct clusters. Unknown data points are classified by evaluating the membership of the k-nearest neighbors based on a distance measure, such as Euclidean distance (59). The algorithm is based on the idea that similar samples lie closer together in the feature space. To achieve results that are more robust to noisy data or imperfect training sets, k was set in this study as k = 5.

(Artificial) neural networks, based on the biological concept of neurons, are formed by a multitude of interconnected artificial neurons (60) taking multiple inputs and outputting the weighted sum after applying an activation function (61). The learning ability is achieved by adapting the neuron’s weights (axons) based on a sequence of training samples during a learning phase. Here, a multilayer neural network was created that contains a single hidden layer with 100 neurons and utilizes the Adam optimizer (62) and a cross-entropy (63) loss function for optimization. The training was performed in batches of 256 samples.

Here, affected datasets were balanced, and training and evaluation were performed using 5-fold cross-validation.

#### Visualization.

Classification accuracies are represented in violin plots showing values of individual cross-validation runs, including median and upper and lower quartiles.

The dendrogram uses the 26 most important features selected by random feature importance, and hierarchical clustering was performed using single linkage (64). Unsupervised UMAP (49) algorithm was applied to visualize the similarity of all data points between different groups as labeled in the respective experiments. Contour lines visualize bivariate kernel density estimation of the underling UMAP samples representing continuous probability density of each cell population in the 2D embedding.

The tumor heterogeneity score is implemented as an indicator for homogeneity/heterogeneity of a cluster calculated by distance of the data points to their respective cluster center. For an individual data point *x_i_*, it was calculated as *d*_i_ = || *x_i_ – c* ||^2^ with *c* being the center of the cluster.

### Statistics

Shapiro-Wilk normality tests were carried out to check datasets for normal distribution. SD where appropriate is indicated in figure legends, and data are shown as mean ± SD or as median with upper and lower quartiles. Unpaired 2-tailed *t* tests and ANOVA were carried out as indicated in figure legends (*P* values are indicated). *P* < 0.05 was considered statistically significant. Dunn’s multiple comparisons test was used to compare the tumor heterogeneity score between different cell lines.

### Study approval

The PDOs were established according to the Declaration of Helsinki and approved by the local ethics committee of TUM School of Medicine and Health (Project 207/15, 946/07, 330/19, and 80/17S). Written informed consent from patients for research was obtained prior to investigation.

### Data availability

Underlying data points are available in the Supporting Data Values document. ScRNA-Seq data are made publicly available via Zenodo at https://doi.org/10.5281/zenodo.14996687 and bulk RNA sequencing data via Zenodo and https://doi.org/10.5281/zenodo.15310375

## Author contributions

KW and MR conceived and designed the study. LF coordinated patient recruitment. KW, CK, AP, LS, AS, CEI, FO, EE, JM, and PW conducted experiments and/or provided experimental support. ML, SR, DEF, FH, NH, and KD performed DHM image and data analysis. NK, CM, DL, SB, RÖ, RR, and BK performed proteomic analysis and RNA sequencing with bioinformatic analysis. IED, HF, TL, and RMS performed endoscopic ultrasound/surgery and provided biopsies and study support. Funding was provided by RMS, RR, DS, GS, BK, OH, KD, and MR. KW and MR wrote the manuscript. All authors reviewed and approved the manuscript.

## Supplementary Material

Supplemental data

Supporting data values

## Figures and Tables

**Table 1:**
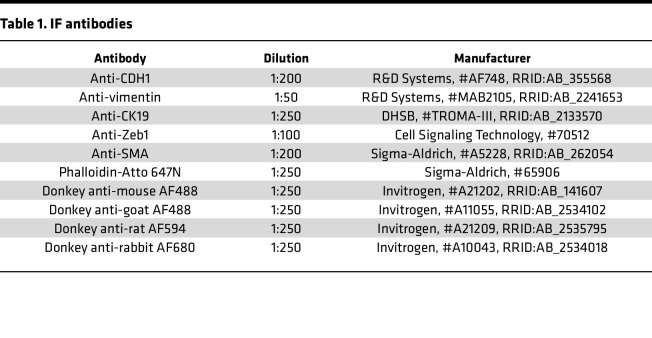
IF antibodies

**Table 2:**
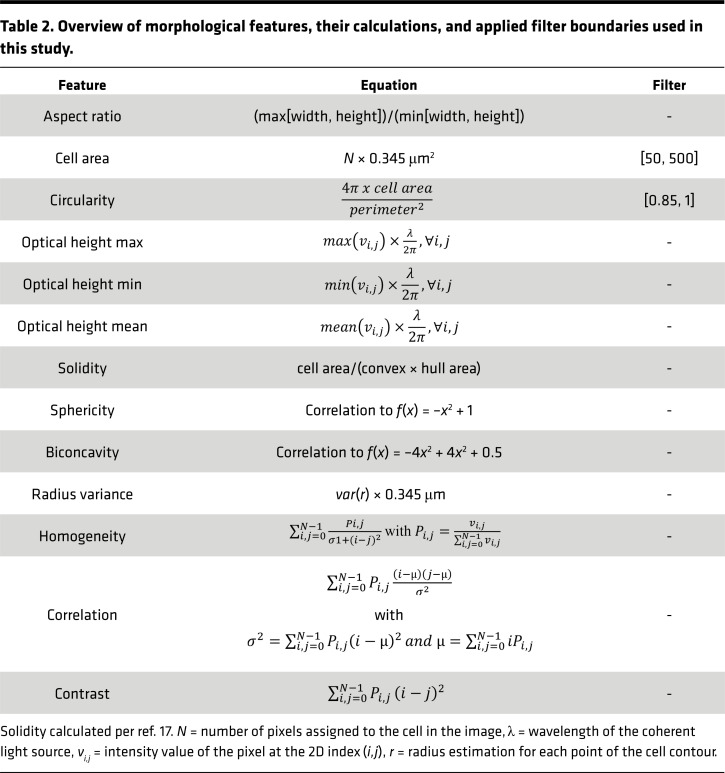
Overview of morphological features, their calculations, and applied filter boundaries used in this study.

## Version 1 05/27/2025

In-Press Preview

## Version 2 07/08/2025

Electronic publication
