# Supplementary material for: Label-free single-cell phenotyping to determine tumor cell heterogeneity in pancreatic cancer in real time
Source: JCI Insight. 2025 May 27;10(13):e169105. doi: 10.1172/jci.insight.169105 (PMC12288891; doi:10.1172/jci.insight.169105)
Supplement: Supplemental data [file jciinsight-10-169105-s062.pdf]

Supplementary Information for

Label-free Single Cell Phenotyping to Determine Tumor Cell Heterogeneity in Pancreatic Cancer in Real-time

Katja Wittenzellner[1,2], Manuel Lengl[3], Stefan Röhrl[3], Carlo Maurer[1,2], Christian Klenk[4], Aristeidis Papargyriou[1,2,5,6,7,8], Laura Schmidleitner[1,2,5,6], Nicole Kabella[9], Akul Shastri[1,2,5,6], David E. Fresacher[3], Farid Harb[3], Nawal Hafez[3], Stefanie Bärthel[10,11,12], Daniele Lucarelli[10,11,12,13], Carmen Escorial-Iriarte[1,4], Felix Orben[1,2], Rupert Öllinger[14], Ellen Emken[4], Lisa Fricke[1,2], Joanna Madej[1,2], Patrick Wustrow[1,2], I. Ekin Demir[15], Helmut Friess[15], Tobias Lahmer[2], Roland M. Schmid[2], Roland Rad[10,13], Günter Schneider[16], Bernhard Kuster[5], Dieter Saur[10,11,12], Oliver Hayden[4], Klaus Diepold[3], Maximilian Reichert[1,2,6,7,8,11]*

[1] Translational Pancreatic Cancer Research Center, TUM School of Medicine and Health, Department of Clinical Medicine – Clinical Department for Internal Medicine II, University Medical Center, Technical University of Munich, 81675 Munich, Germany

[2] TUM School of Medicine and Health, Department of Clinical Medicine – Clinical Department for Internal Medicine II, University Medical Center, Technical University of Munich

[3] Chair for Data Processing, Technical University of Munich, 80333 Munich, Germany

[4] Heinz Nixdorf Chair of Biomedical Electronics, TranslaTUM, Technical University of Munich, 81675 Munich, Germany

[5] Center for Protein Assemblies (CPA), Technical University of Munich, 85747 Garching, Germany.

[6] Center for Organoid Systems (COS), Technical University Munich (TUM), 85747 Garching, Germany.

[7] Bavarian Cancer Research Center (BZKF), Munich, Germany

[8] Institute of Stem Cell Research, Helmholtz Center Munich, 85764 Neuherberg, Germany

[9] Chair of Proteomics and Bioanalytics, Technical University of Munich, 85354 Freising, Germany

[10] Institute for Translational Cancer Research and Experimental Cancer Therapy, TranslaTUM, Technical University of Munich, 81675 Munich, Germany

[11] German Cancer Consortium (DKTK), partner site Munich, a partnership between DKFZ and University Hospital Klinikum rechts der Isar, Germany

[12] German Cancer Research Center (DKFZ) and German Cancer Consortium (DKTK), 69120 Heidelberg, Germany

[13] Department of Computational Health, Institute of Computational Biology, Helmholtz, Munich, Germany

[14] Institute of Molecular Oncology and Functional Genomics, TranslaTUM, TUM School of Medicine, Technical University of Munich, 81675 Munich, Germany

[15] Department of Surgery, TUM Universitätsklinikum, Klinikum rechts der Isar, Technical University of Munich, 81675 Munich, Germany

[16] University Medical Center Göttingen, Department of General, Visceral and Pediatric Surgery, 37075 Göttingen, Germany

* Corresponding author: Maximilian Reichert, Medical Clinic and Polyclinic II, Klinikum rechts der Isar, Technical University Munich, 81675 München, Germany, e-mail: maximilian.reichert@tum.de

This supplement includes:

- Supplementary Figures 1-8
- Legends for Dataset S1-12

Other supporting materials for this manuscript include the following:

- Datasets S1-S12

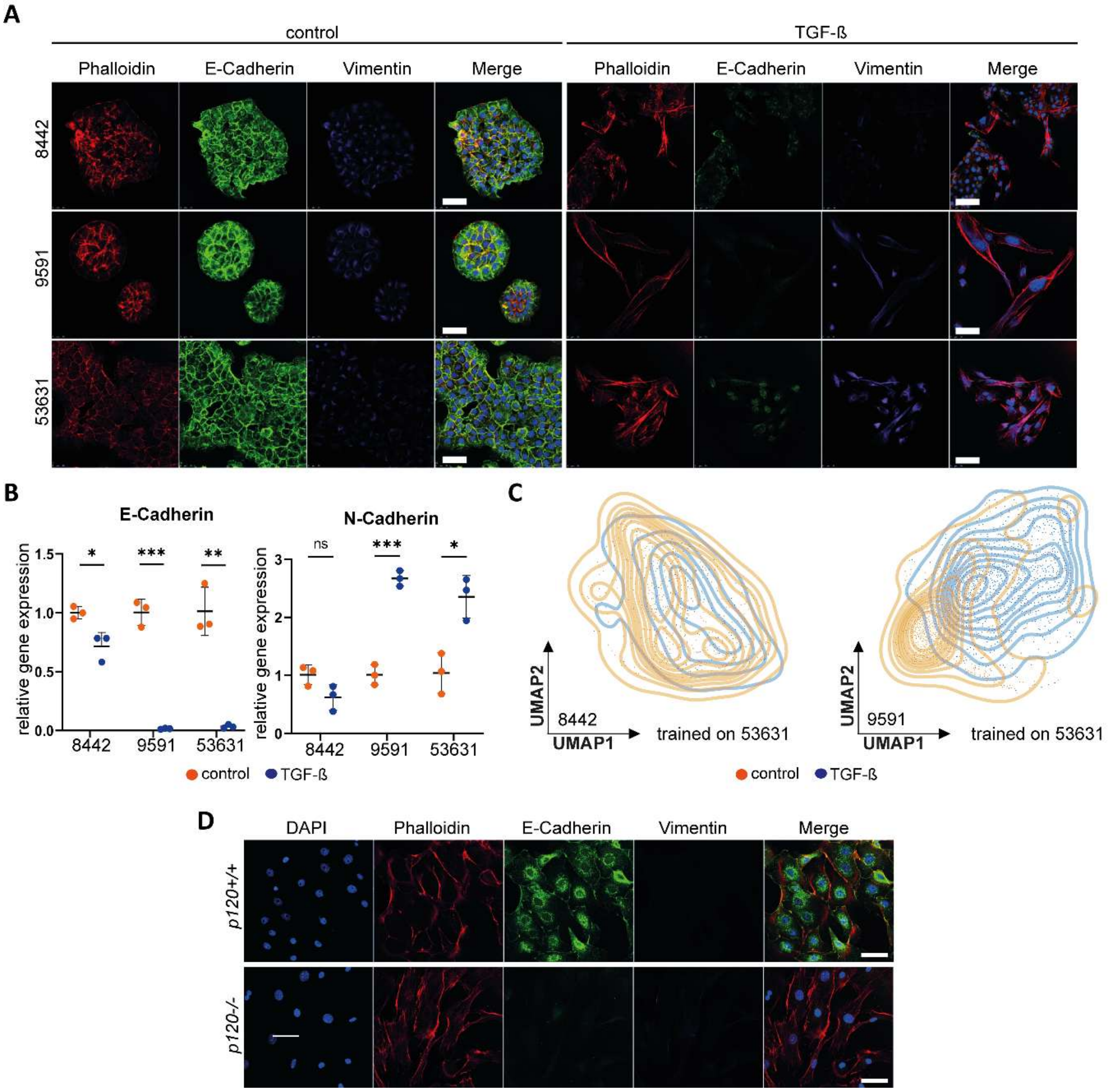

Supplementary Figure 1: Validation of pharmacologic and genetic induced EMT.

A, Immunofluorescence staining of F-Actin (phalloidin, red), E-Cadherin (green) and Vimentin (purple) in control and TGF-β-treated cells. Scale bars represent 100 µm.

B, Relative mRNA expression levels of E-Cadherin and N-Cadherin in the control and TGF-β-treated cells. Shown is the mean ± SD of three independent experiments. Unpaired student's t test: ns = not significant, * $p<0.05$, **$p<0.01$, and ***$p<0.001$.

C, Unsupervised UMAP clustering of the control and TGF-β-treated cells of lines 8442 and 9591 tested with the algorithm trained on the control cells of line 53631.

D, Immunofluorescence staining of DAPI (blue), F-Actin (phalloidin, red), E-Cadherin (green) and Vimentin (purple) in p120+/+ (p120-catenin wildtype) and p120-/- (p120-catenin knockout) cells. Scale bar indicates 100 µm.

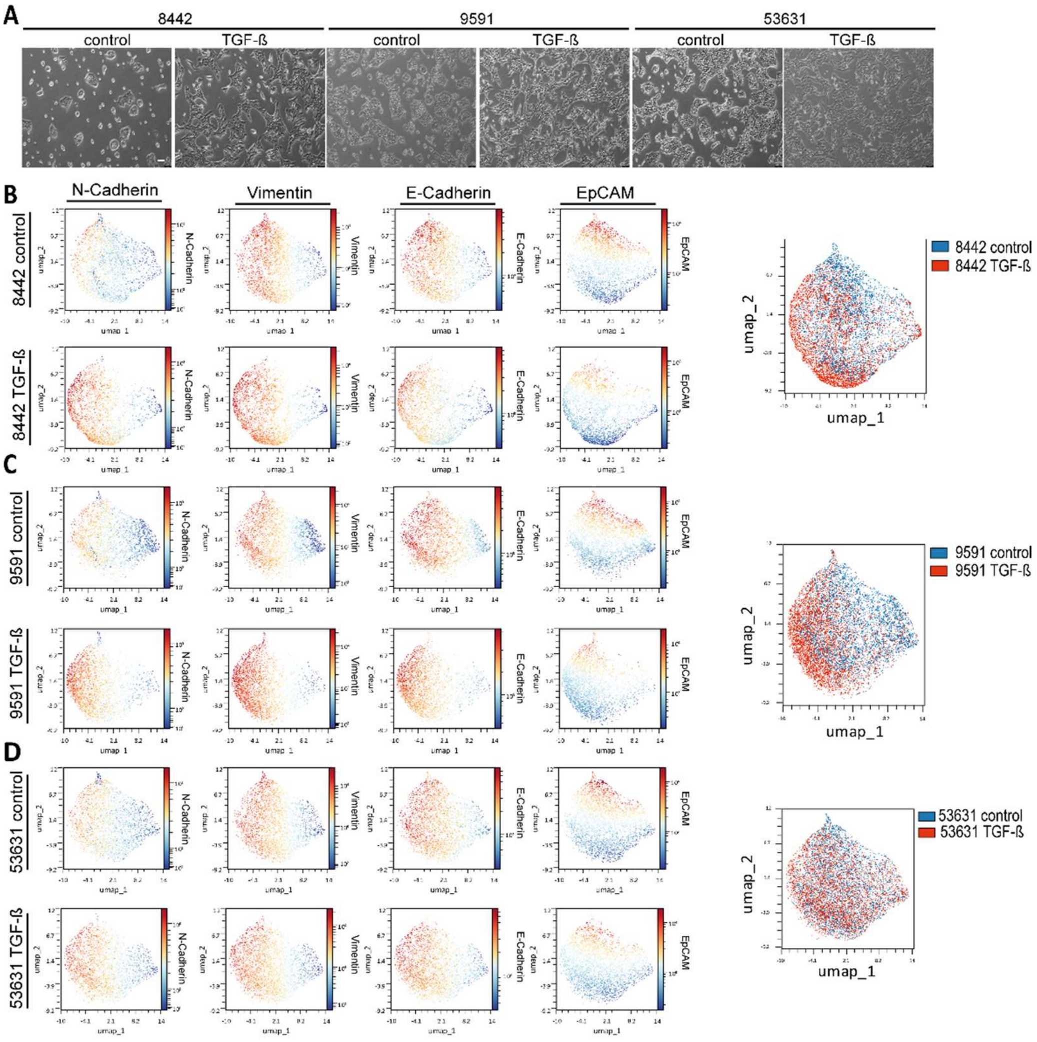

Supplementary Figure 2: Intra-cell line heterogeneity in murine PDAC cells treated with TGF- β analyzed using flow cytometry.

A, Phase contrast images of control and TGF-β-treated epithelial PDAC cells. Scale bars represent 200 µm.

B-D, Flow cytometry analysis of the EMT markers N-Cadherin, Vimentin, E-Cadherin and EpCAM in cell lines 8442 (B), 9591 (C) and 53631 (D) in the control versus TGF- β-treated condition visualized as UMAP plots. Right UMAP plots demonstrate the overall shift in phenotype upon TGF- β treatment of all four markers together.

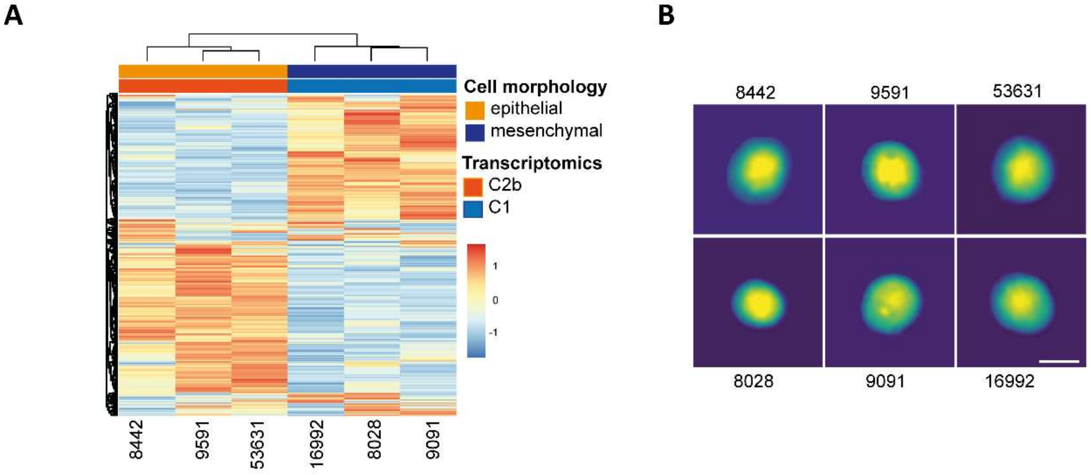

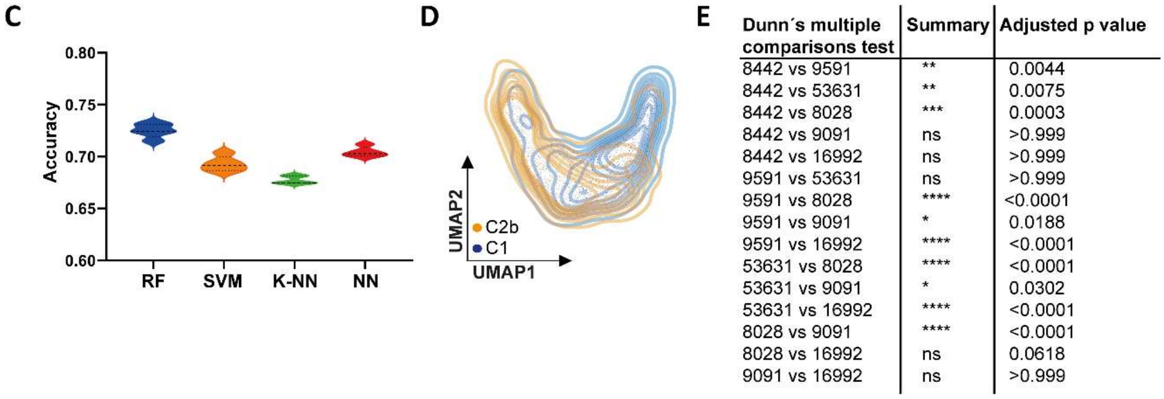

E

Dunn´s multiple comparisons test	Summary	Adjusted p value
8442 vs 9591	**	0.0044
8442 vs 53631	**	0.0075
8442 vs 8028	***	0.0003
8442 vs 9091	ns	>0.999
8442 vs 16992	ns	>0.999
9591 vs 53631	ns	>0.999
9591 vs 8028	****	<0.0001
9591 vs 9091	*	0.0188
9591 vs 16992	****	<0.0001
53631 vs 8028	****	<0.0001
53631 vs 9091	*	0.0302
53631 vs 16992	****	<0.0001
8028 vs 9091	****	<0.0001
8028 vs 16992	ns	0.0618
9091 vs 16992	ns	>0.999

Supplementary Figure 3: Inter- and intra-cell line heterogeneity in murine PDAC cells.

A, Hierarchical clustering of the 2000 most variable genes based on transcriptomic profiles of the six cell lines averaged over three independent replicates per cell line.

B, Representative DHM phase images in false colors of PDAC cells in suspension. Scale bar indicates 10 µm.

C, Accuracy for separating epithelial and mesenchymal PDAC cells using different classification methods: random forest (RF), support vector machine (SVM), K-nearest neighbors (K-NN) and neural network (NN). Shown are the median, upper, and lower quartiles.

D, Unsupervised clustering of DHM phase images derived from cells of the C2b (epithelial) and the C1 (mesenchymal) cluster visualized using the UMAP plot.

E, Dunn's multiple comparisons test compared differences between murine PDAC lines. ns = not significant, * p<0.05, **p<0.01, ***p<0.001, ****p<0.0001.

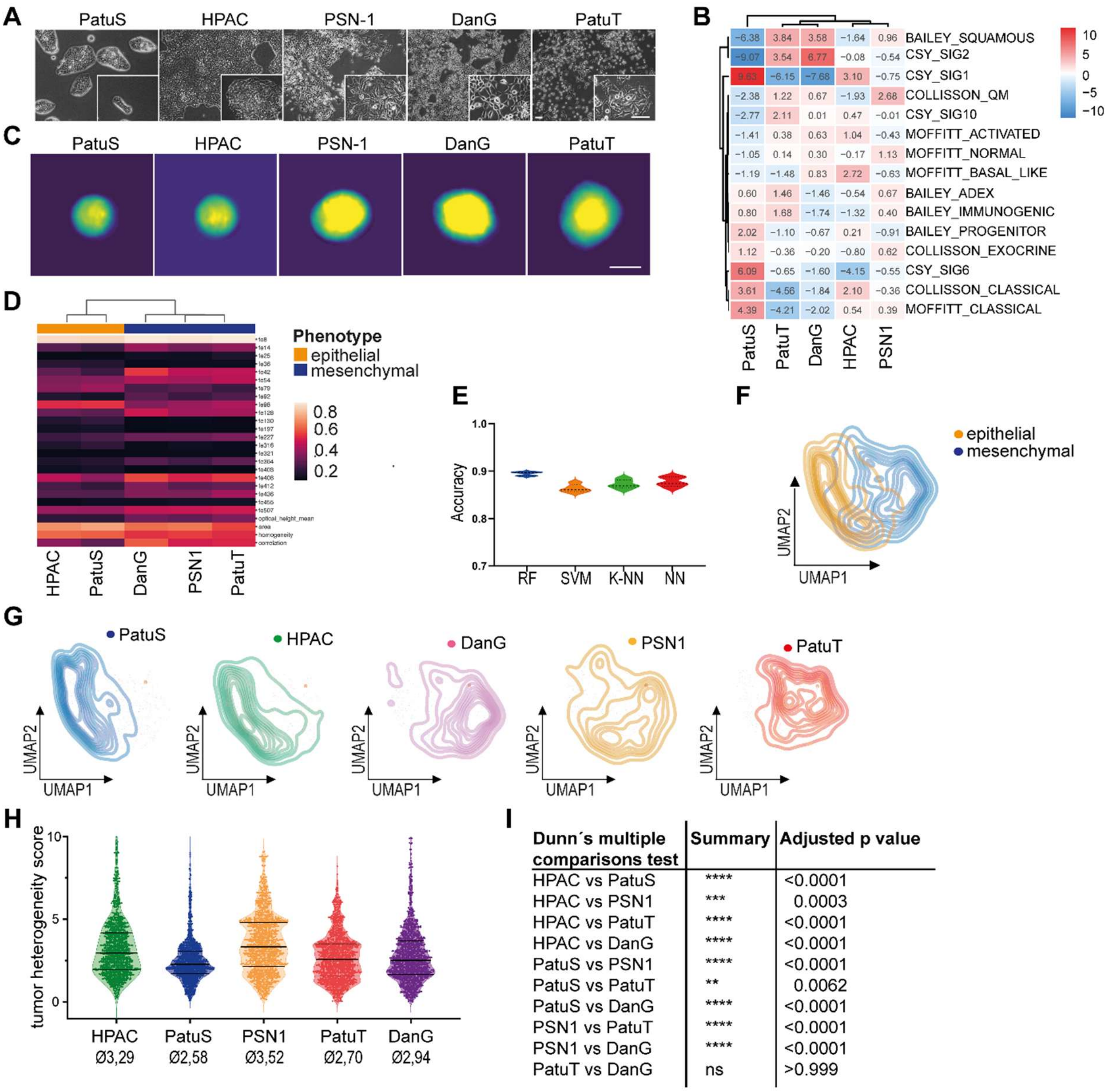

Supplementary Figure 4 Single cell phenotyping of human characterized PDAC cells using DHM.

A, Phase contrast images of human established PDAC cells. Scale bars represent 200 µm.

B, Molecular subtype classifier gene sets applied to transcriptomic profiles of human PDAC cells.

C, Representative DHM phase images in false colors of human established PDAC cells in suspension. Scale bar indicates 10 µm.

D, Hierarchical clustering of DHM phase images derived from human established PDAC cells based on the most different Resnet18 and morphological features.

E, Accuracy for separating human epithelial and quasi-mesenchymal PDAC cells using different classification methods: random forest (RF), support vector machine (SVM), K-nearest neighbors (K-NN) and neural network (NN). Shown are the median, upper, and lower quartiles.

F, Unsupervised clustering of PatuS and HPAC (epithelial) versus PSN1, DanG and PatuT (mesenchymal) based on DHM phase images and visualized using the UMAP plot.

G, Unsupervised clustering of individual cell lines based on DHM phase images and visualized using UMAP plots.

H, Evaluation of intra-cell line heterogeneity using single cell distance to cluster centroid. Kruskal-Wallis test, ****$p<0.0001$. Shown are the median, upper, and lower quartiles.

I, Dunn's multiple comparisons test compared differences between human PDAC lines. ns = not significant, **$p<0.01$, ***$p<0.001$, ****$p<0.0001$.

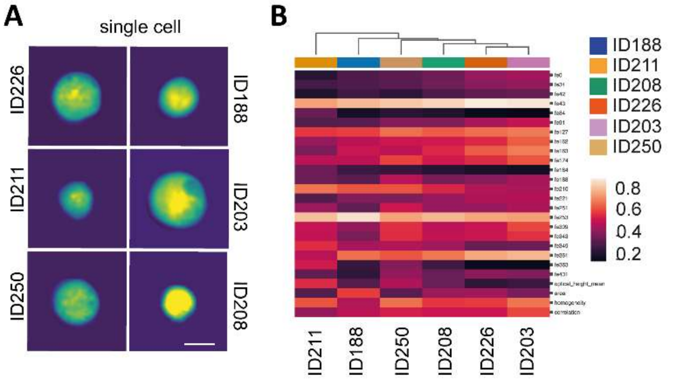

Dunn´s multiple comparisons test	Summary	Adjusted p value
ID188 vs ID211	*	0.0122
ID188 vs ID226	ns	>0.999
ID188 vs ID250	ns	>0.999
ID188 vs ID203	ns	>0.999
ID188 vs ID208	*	0.0167
ID211 vs ID226	ns	0.6162
ID211 vs ID250	**	0.0031
ID211 vs ID203	ns	0.0633
ID211 vs ID208	****	<0.0001
ID226 vs ID250	ns	>0.999
ID226 vs ID203	ns	>0.999
ID226 vs ID208	****	<0.0001
ID250 vs ID203	ns	>0.999
ID250 vs ID208	ns	0.0884
ID203 vs ID208	**	0.0025

Supplementary Figure 5: DHM-based phenotyping of human patient-derived organoids.

A, Representative single cell DHM phase images in false colors of patient-derived PDAC organoids in suspension. Scale bar indicates 10 µm.

B, Hierarchical clustering of patient-derived PDAC organoids based on most differential morphological and Resnet18 features derived from single cell phase images.

C, Dunn's multiple comparisons test compared differences between PDO lines. ns = not significant, *p<0.05, **p<0.01, ****p<0.0001.

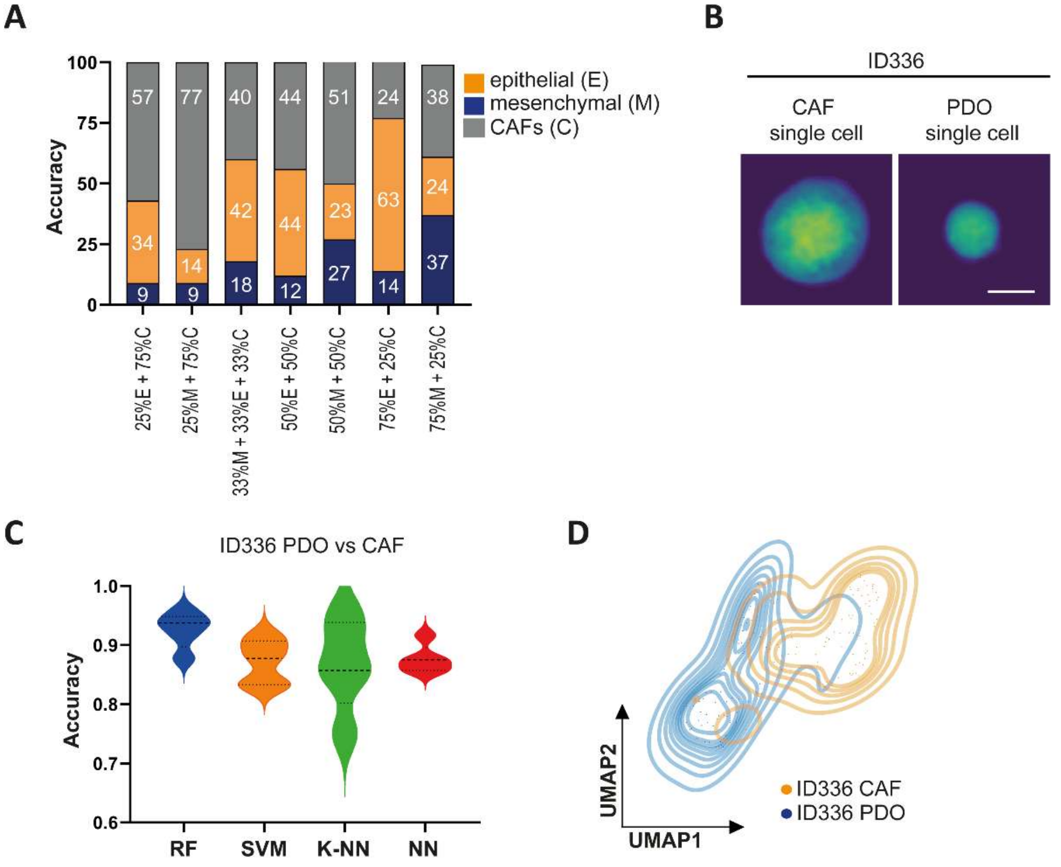

Supplementary Figure 6: Distinguishing different tumor cell populations and cancer-associated fibroblasts using DHM.

A, Accuracies in samples with mixed cell types obtained using random forest classification when trained with 100% epithelial (9591), 100% mesenchymal (16992) PDAC cells and murine 100% CAFs.

B, Representative single cell DHM phase images in false colors of matched patient-derived PDAC organoids (PDO) and cancer-associated fibroblasts (CAF) in suspension. Scale bar indicates 10 µm.

C, Accuracy for separating matched patient-derived organoids and CAFs using different classification methods: random forest (RF), support vector machine (SVM), K-nearest neighbors (K-NN) and neural network (NN). Shown are the median, upper, and lower quartiles.

D, Unsupervised clustering of matched ID336 PDO versus ID336 CAF based on DHM phase images and visualized using the UMAP plot.

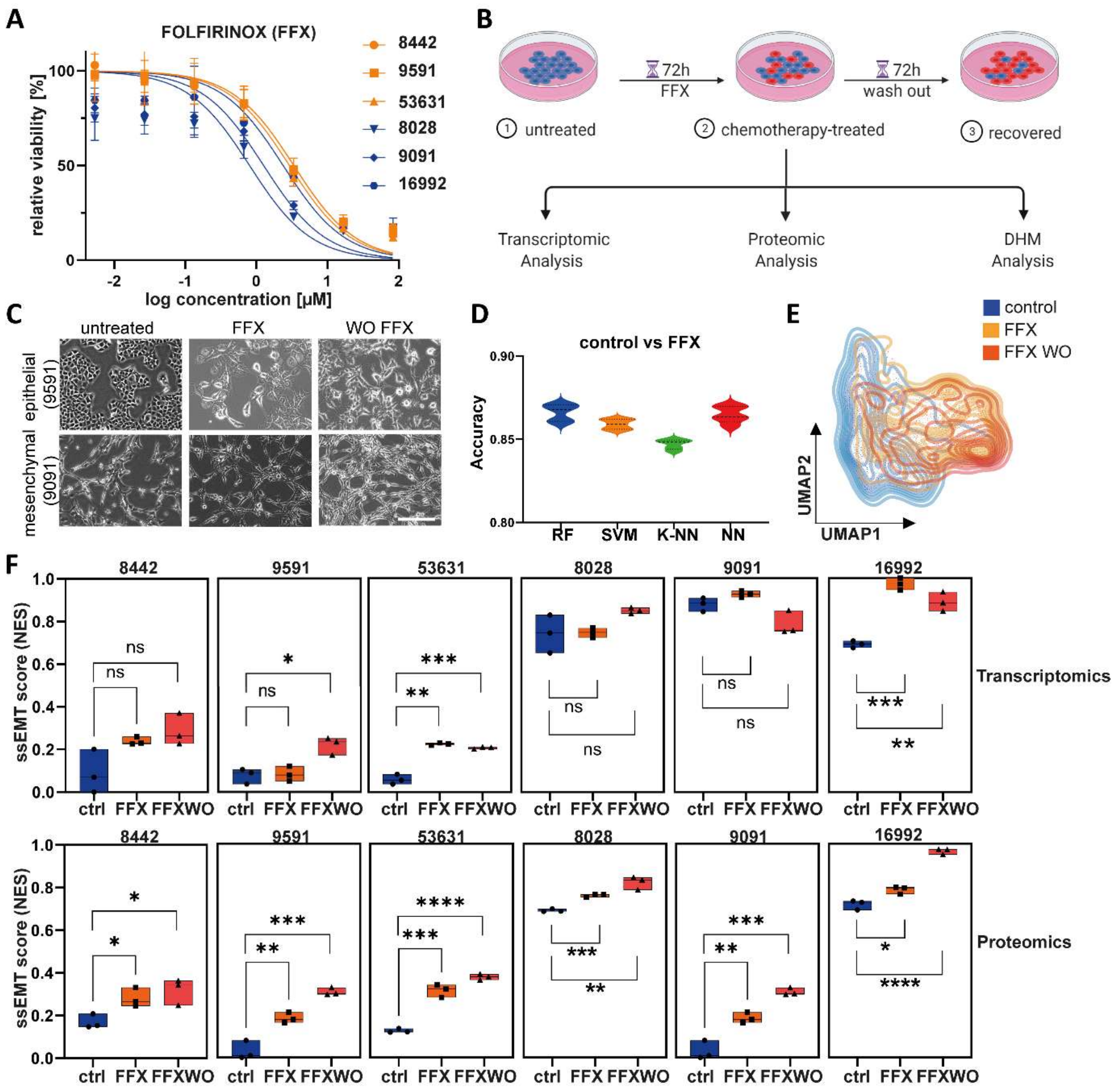

Supplementary Figure 7: Phenotype changes upon FOLFIRINOX treatment in murine PDAC cells.

A, Dose–response curves of murine PDAC cells treated with FOLFIRINOX (FFX) over three days. Shown is the mean ± SD of three independent experiments.

B, Schematic illustration of the experimental workflow.

C, Representative images of epithelial and mesenchymal cells in the untreated, FFX and washout condition. Scale bar indicates 200µm.

D, Accuracy for separating untreated versus FFX treated PDAC cells using different classification methods: random forest (RF), support vector machine (SVM), K-nearest neighbors (K-NN) and neural network (NN). Shown are the median, upper, and lower quartiles.

E, Unsupervised clustering of all murine PDAC cells combined in an untreated, FFX treated or FFX washout condition based on DHM phase images and visualized using the UMAP plot.

F, Single-sample normalized enrichment scores (NES) of the HALLMARK_EPITHELIAL_ MESENCHYMAL_TRANSITION gene set (ssEMT score) retrieved from RNA sequencing (upper panel) and proteomic analysis (lower panel). Shown is the median of three independent replicates per cell line. Unpaired student's t test: ns = not significant, *p<0.05, **p<0.01, ***p<0.001 and ****p<0.0001.

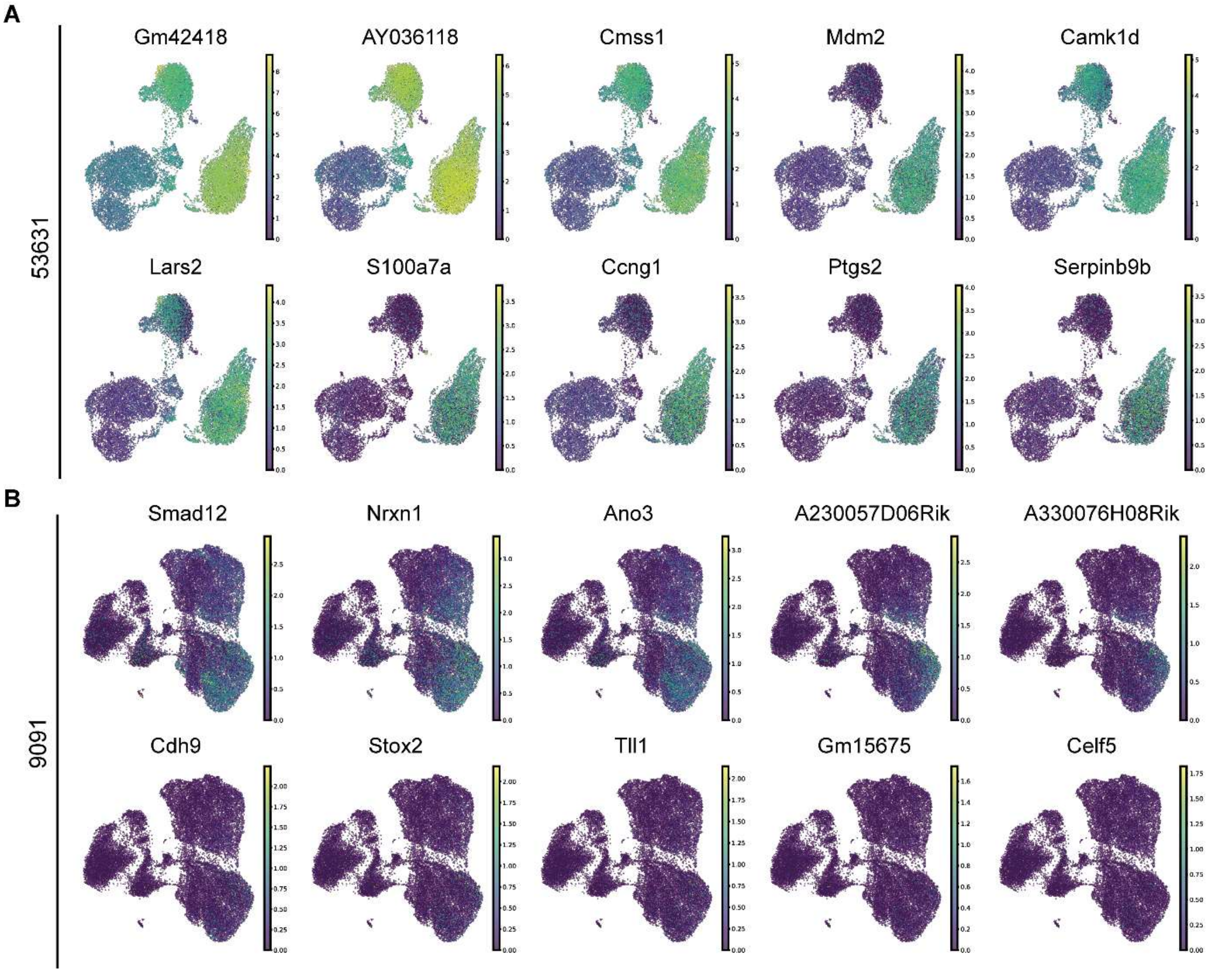

Supplementary Figure 8: Single cell RNA sequencing of murine PDAC cells upon FOLFIRINOX treatment.

A, Top ten upregulated genes in cell line 53631 upon FOLFIRINOX treatment visualized in UMAP plots illustrating gene expression intensity.

B, Top ten upregulated genes in cell line 9091 upon FOLFIRINOX treatment visualized in UMAP plots illustrating gene expression intensity.

Dataset document including:

Data file 1. Relative mRNA expression levels of E-Cahderin and N-Cadherin in the control and TGF-ß treated PDAC cells.

Data file 2: Accuracy for separating control and TGF-β-treated PDAC cells individually for every cell line using different classification methods: random forest (RF), support vector machine (SVM), K-nearest neighbors (K-NN) and neural network (NN).

Data file 3: Accuracy for separating p120+/+ and p120-/- cells using different classification methods: random forest (RF), support vector machine (SVM), K-nearest neighbors (K-NN) and neural network (NN).

Data file 4: Accuracy for separating epithelial (C2b) and mesenchymal (C1) PDAC cells using different classification methods: random forest (RF), support vector machine (SVM), K-nearest neighbors (K-NN) and neural network (NN).

Data file 5. Tumor heterogeneity score of murine PDAC cells calculated as the single cell distance to cluster centroid.

Data file 6: Accuracy for separating human epithelial and quasi-mesenchymal PDAC cells using different classification methods: random forest (RF), support vector machine (SVM), K-nearest neighbors (K-NN) and neural network (NN).

Data file 7. Tumor heterogeneity score of human PDAC cells calculated as the single cell distance to cluster centroid.

Data file 8. Tumor heterogeneity score of human patient-derived organoids calculated as the single cell distance to cluster centroid.

Data file 9: Accuracy for separating matched patient-derived organoids and CAFs using different classification methods: random forest (RF), support vector machine (SVM), K-nearest neighbors (K-NN) and neural network (NN).
Data file 10: Accuracy for separating untreated versus FFX treated PDAC cells using different classification methods: random forest (RF), support vector machine (SVM), K-nearest neighbors (K-NN) and neural network (NN).

"Data file 11. Single-sample normalized enrichment scores (NES) of the HALLMARK_EPITHELIAL_ MESENCHYMAL_TRANSITION gene set (ssEMT score) retrieved from RNA sequencing

and proteomic analysis "

Data file 12. Tumor heterogeneity score of murine PDAC cells upon FOLFIRINOX treatment (FFX) and a wash out phase (WO) calculated as the single cell distance to cluster centroid.

Data file 13: Accuracy for separating organoids pre (ID188) and post (ID211) FOLFIRINOX treatment using different classification methods: random forest (RF), support vector machine (SVM), K-nearest neighbors (K-NN) and neural network (NN).

Data file 14. Tumor heterogeneity score of human patient-derived organoids ID188 versus ID211 calculated as the single cell distance to cluster centroid.
